# Effects of adult temperature on gene expression in a butterfly: identifying pathways associated with thermal acclimation

**DOI:** 10.1186/s12862-019-1362-y

**Published:** 2019-01-23

**Authors:** Kristin Franke, Isabell Karl, Tonatiuh Pena Centeno, Barbara Feldmeyer, Christian Lassek, Vicencio Oostra, Katharina Riedel, Mario Stanke, Christopher W. Wheat, Klaus Fischer

**Affiliations:** 1 Zoological Institute and Museum, University of Greifswald, D-17489 Greifswald, Germany; 2 Institute for Mathematics and Computer Science, University of Greifswald, D-17487 Greifswald, Germany; 3 Senckenberg Biodiversity and Climate Research Centre (BiK-F), Molecular Ecology Group, D-60325 Frankfurt am Main, Germany; 4 Institute for Microbiology, University of Greifswald, D-17489 Greifswald, Germany; 5 Department of Genetics, Evolution and Environment, University College London, WC1E 6BT, London, UK; 6 Zoological Institute, Stockholm University, SE-10691 Stockholm, Sweden; 7 Present address: Institute for Integrated Natural Sciences, University Koblenz-Landau, Universitätsstraße 1, D-56070 Koblenz, Germany

**Keywords:** *Bicyclus anynana*, Heat tolerance, Oxidative stress, Phenotypic plasticity, RNAseq, Transcriptome

## Abstract

**Background:**

Phenotypic plasticity is a pervasive property of all organisms and considered to be of key importance for dealing with environmental variation. Plastic responses to temperature, which is one of the most important ecological factors, have received much attention over recent decades. A recurrent pattern of temperature-induced adaptive plasticity includes increased heat tolerance after exposure to warmer temperatures and increased cold tolerance after exposure to cooler temperatures. However, the mechanisms underlying these plastic responses are hitherto not well understood. Therefore, we here investigate effects of adult acclimation on gene expression in the tropical butterfly *Bicyclus anynana*, using an RNAseq approach.

**Results:**

We show that several antioxidant markers (e.g. *peroxidase*, *cytochrome P450*) were up-regulated at a higher temperature compared with a lower adult temperature, which might play an important role in the acclamatory responses subsequently providing increased heat tolerance. Furthermore, several metabolic pathways were up-regulated at the higher temperature, likely reflecting increased metabolic rates. In contrast, we found no evidence for a decisive role of the heat shock response.

**Conclusions:**

Although the important role of antioxidant defence mechanisms in alleviating detrimental effects of oxidative stress is firmly established, we speculate that its potentially important role in mediating heat tolerance and survival under stress has been underestimated thus far and thus deserves more attention.

**Electronic supplementary material:**

The online version of this article (10.1186/s12862-019-1362-y) contains supplementary material, which is available to authorized users.

## Background

Most organisms are faced with heterogeneous and fluctuating environmental conditions, warranting the capability to adjust the expression of phenotypes to prevailing environmental conditions [1, 2]. Such adjustments may result from genetic adaptation or phenotypic plasticity, with the latter comprising a fast and effective means to alter fitness-related traits [3, 4]. Consequently, adaptive plastic responses are considered to be an important aspect of population adaptation [4, 5]. Temperature is an important environmental factor warranting phenotypic adjustment [4, 5]. In butterflies, for instance, temperature-induced adaptive plasticity has been found in a variety of traits including temperature stress resistance [6–11]. Regarding the latter, there is evidence across several insect taxa that cold tolerance increases after exposure to cool and heat tolerance after exposure to warm temperatures [9, 10, 12, 13]. In recent years, temperature-mediated plasticity in stress tolerance has gained renewed interest, owing to concerns about the potential impact of anthropogenic climate change on extant biodiversity [4, 5, 14]. While phenotypic plasticity may be induced during development or in the adult stage, we here exclusively focus on acclimation to different adult temperatures [10, 15–20]. This was motivated by the facts that adult temperature induces clear variation in thermal stress tolerance, and in order to exclude potentially confounding effects of the developmental environment.

To gain a more complete understanding of the prospects and limitations of beneficial acclimation in buffering effects of temperature variation, a better understanding of the underlying mechanisms is important. At the mechanistic level, phenotypic plasticity in thermal tolerance may be caused by environmental effects on gene and protein expression or biosynthetic pathways [21, 22], but currently our understanding of the underlying mechanisms is very limited [15, 23–27]. Recent developments in sequencing techniques though have opened up new opportunities to address this issue, enabling unbiased approaches by targeting entire genomes and transcriptomes [15, 28]. Against this background, we here apply an RNAseq approach to identify pathways affected by adult temperature, which in turn may indicate the mechanisms causing phenotypic changes during acclimation, in the tropical butterfly *Bicyclus anynana*.

Since we are interested in adult acclimation here, all individuals were reared in a common environment. To subsequently induce acclimation responses, adult butterflies were exposed to different temperatures and also feeding treatments. Including the latter factor was motivated by the facts that (1) in nature animals are frequently faced with transient periods of food shortage negatively affecting fitness components (e.g. [29, 30]), and as (2) plasticity is assumed to involve costs such that inadequate nutrition may interfere with plastic responses [5]. According trade-offs though may be masked by having food access ad libitum. We deliberately decided to use non-stressful temperatures (19 and 27 °C) to induce acclamatory responses, as we were interested in the mechanisms conferring increased heat tolerance after acclimation rather than the stress response per se which is reasonably well understood (e.g. [26]). The temperatures chosen are well within the range of temperature fluctuations typically experienced by adult butterflies in their natural environment within their life spans. Additionally, we explore sexual differences in gene expression, as males and females typically differ in their responses to environmental variation (e.g. [31, 32]). Our study organism seems to be eminently suitable for our purpose as it inhabits a highly seasonal environment, thus relying heavily on phenotypic plasticity to master associated challenges [33]. Moreover, plastic responses to adult acclimation temperatures and feeding regimes have been described in detail. For instance, exposing adult butterflies for a few days to the temperatures used here results in an increase in heat tolerance by ca. 60%, a decrease in cold tolerance by ca. 36%, an increase in fecundity (ca. 22%) but a decrease in egg size (ca. 15%) at the higher compared with the lower temperature [4, 6, 8, 30, 34–36].

Our principle aim here is to identify pathways that are affected by adult temperature and which may therefore be mechanistically linked to the increased heat tolerance after acclimation to higher temperatures [10]. Specifically, we address the following hypotheses: (1) Temperature differences, although exclusively experienced in the adult stage, will affect expression profiles. While much of this variation will be attributable to changes in metabolism, we will specifically focus on pathways that may affect heat tolerance. Namely, an upregulation of the heat shock response and of oxidative defense mechanisms at the higher temperature is expected [37, 38]. (2) Owing to costs of plasticity, food stress will interfere with plastic responses as evidenced by the occurrence of temperature by feeding treatment interactions. Additionally, we explore sexual differences in the response to temperature, evidenced by the occurrence of temperature by sex interactions.

## Results

### Variation in life-history and physiological traits

**Fig. 1:**
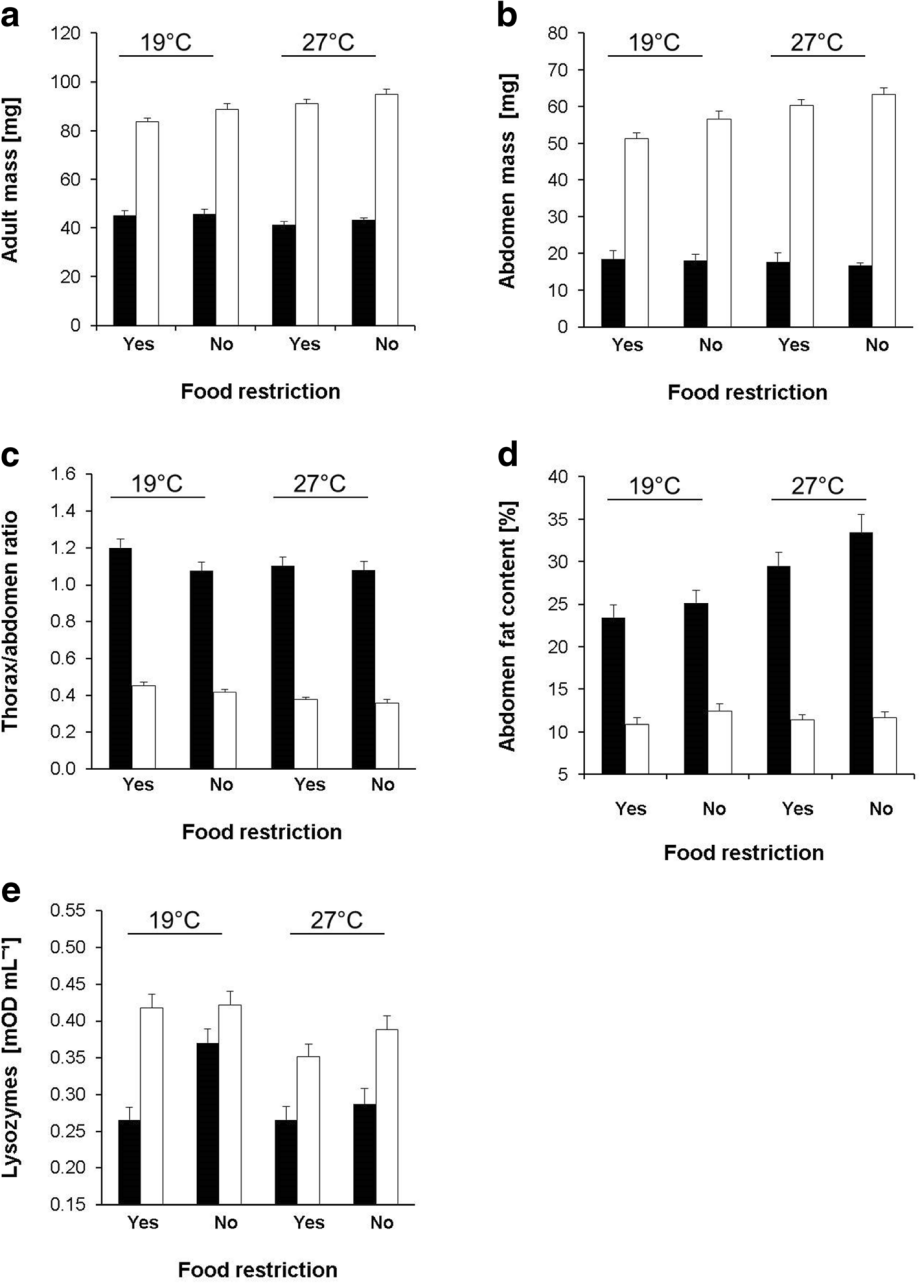
Trait variation in *Bicyclus anynana* butterflies across adult feeding treatments and two temperatures. Given are means + 1 SE for (**a**) total body mass, (**b**) abdomen mass, (**c**) thorax-abdomen ratio, (**d**) abdomen fat content, and (**e**) lysozyme activity for 8-day old adults. Food restriction = yes: only 30 min access to food per 48 h; food restriction = no: food access ad libitum throughout. Black bars: males; open bars: females. Group sample sizes range between 33 and 48

Our treatments were effective in manipulating phenotypic values. Adult temperature significantly affected thorax mass, abdomen mass, and thorax-abdomen ratio, but not total adult mass (see Table 1 for all statistical results). The lower compared with the higher adult temperature caused higher thorax (20.1 ± 0.3 mg > 19.6 ± 0.3 mg) but lower abdomen masses (37.4 ± 1.7 mg < 42.8 ± 1.9 mg), and higher thorax-abdomen ratios (0.76 ± 0.03 > 0.68 ± 0.03) owing to much larger temperature-induced variation in abdomen than in thorax mass (Fig. 1). All four traits measured differed significantly among sexes, indicating that males compared with females had lower adult body (43.8 ± 0.9 mg < 89.8 ± 1.1 mg), thorax (17.3 ± 0.2 mg < 21.9 ± 0.2 mg) and abdomen masses (17.8 mg ± 1.0 mg < 58.0 mg ± 1.0 mg) but a higher thorax-abdomen ratio (1.12 ± 0.02 > 0.40 ± 0.01). Sexual differences in body and abdomen mass were more pronounced at the higher than at the lower temperature (significant temperature by sex interactions). Adult feeding treatment only affected the thorax-abdomen ratio significantly, being higher in food-restricted than in control animals (0.72 ± 0.03 > 0.69 ± 0.03). Family effects were significant for thorax and abdomen mass, though variance component analyses revealed that they explained less than 0.01% of the variation.

**Table 1:** ANOVA results for variation in life history and physiological traits in *Bicyclus anynana*

Trait	Source	*MS*	*DF*	*F*	*P*
Adult mass	Temperature	< 0.001	1	0.6	0.439
	Food	< 0.001	1	2.1	0.144
	Sex	0.166	1	389.4	**< 0.001**
	Temp. * Sex	0.002	1	4.2	**0.040**
	Family (Random)	0.001	9	1.6	0.104
	Error	< 0.001	431		
Thorax mass	Temperature	< 0.001	1	6.4	**0.012**
	Food	< 0.001	1	0.4	0.536
	Sex	0.002	1	420.3	**< 0.001**
	Family (Random)	< 0.001	9	10.9	**< 0.001**
	Error	< 0.001	432		
Abdomen mass	Temperature	0.001	1	6.9	**0.009**
	Food	< 0.001	1	1.8	0.177
	Sex	0.125	1	820.6	**< 0.001**
	Temp. * Sex	0.002	1	11.0	**0.001**
	Family (Random)	< 0.001	9	1.9	**0.046**
	Error	< 0.001	335		
Thorax /Abdomen	Temperature	0.30	1	6.7	**0.010**
	Food	0.21	1	4.7	**0.031**
	Sex	40.27	1	894.8	**< 0.001**
	Family (Random)	0.05	9	1.2	0.309
	Error	0.05	336		
Fat content	Temperature	1201	1	21.6	**< 0.001**
	Food	203	1	3.7	0.057
	Sex	22,346	1	402.3	**< 0.001**
	Temp. * Sex	1267	1	22.8	**< 0.001**
	Family (Random)	193	9	3.5	**< 0.001**
	Error	56	329		
Lysozyme	Temperature	0.17	1	10.1	**0.002**
	Food	0.20	1	11.9	**0.001**
	Sex	1.27	1	77.6	**< 0.001**
	Family (Random)	0.03	9	1.7	0.082
	Error	0.02	417		
ADH	Temperature	0.0002	1	2.6	0.106
	Food	< 0.0001	1	< 0.1	0.885
	Sex	0.0011	1	18.5	**< 0.001**
	Family (Random)	0.0002	9	2.8	**0.004**
	Error	0.0001	432		
Protein	Temperature	0.0005	1	0.7	0.411
	Food	< 0.0001	1	0.1	0.823
	Sex	0.0001	1	0.2	0.687
	Family (Random)	0.0005	9	0.7	0.723
	Error	0.0007	423		

Shown are the effects of adult temperature, adult feeding treatment, sex (fixed factors), and family (random factor). Minimum adequate models were constructed by sequentially removing non-significant interaction terms. Significant *P*-values are given in bold

For physiological traits, adult temperature significantly affected fat content and lysozyme activity only, with fat content being higher at the higher temperature (20.1 ± 1.0% > 17.7 ± 0.8%) while lysozyme activity was higher at the lower temperature (0.37 ± 0.01 mOD/mL > 0.33 ± 0.01 mOD / mL; Table 1, Fig. 1). The only significant interaction was the one between temperature and sex for fat content, indicating that temperature effects were largely restricted to males (Fig. 1d). Differences between sexes were significant for all physiological traits except protein content. Females compared with males had higher lysozyme (0.39 ± 0.01 mOD/mL > 0.30 ± 0.01 mOD/mL) and ADH (0.023 ± 0.001 mOD/mL > 0.021 ± 0.001 mOD/mL) activities, but a lower relative fat content (11.6 ± 0.4% < 27.6 ± 0.9%). Feeding treatment significantly affected lysozyme activity only, showing that control individuals had a higher lysozyme activity than food restricted ones (0.37 ± 0.01 mOD/mL > 0.33 ± 0.01 mOD / mL). Additionally, there was a non-significant tendency towards a higher fat content in control individuals (19.5 ± 0.8% vs. 18.2 ± 0.9%). Family effects were significant for fat content and ADH activity only, but again explained only a minor fraction of the total variation (4.1 and 0.01%, respectively).

### Gene expression

**Fig. 2:**
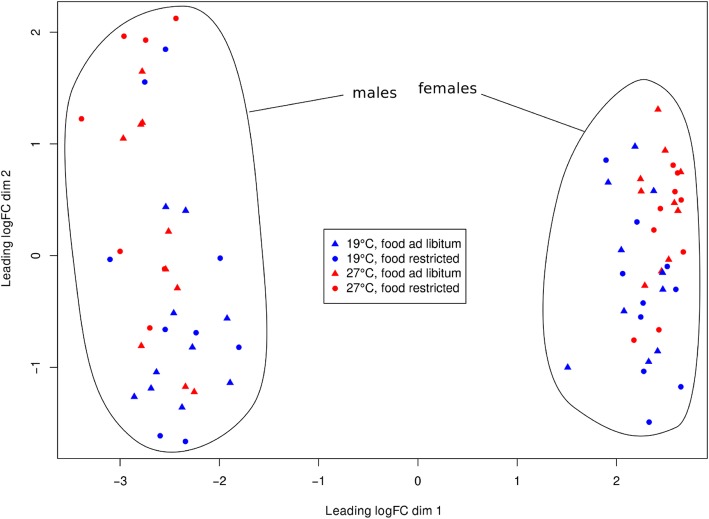
Multidimensional Scaling plot of the expression profiles of all 75 individuals. The sexes are clearly divided and the expression profiles of females appear less diverse than those of males. Moreover, temperature (different colours) seems to have an impact, while an overall effect of feeding treatment (shape) is not apparent

The 10,696 clusters of assembled transcripts mapped primarily to genes known from other insects (99.9% in total), especially to those from other Lepidoptera (93.4%; Additional file 1: Table S1). Searching the BUSCO database (version 9) for insect universal single-copy orthologs in our assembled transcriptome, identified 92.3% of families as at last once completely present, 51.6% as duplicated, 5.4% as fragmented, and 2.3% as missing. These results indicate that a large fraction of universally present insect genes are also represented in our assembled transcriptome. The large number of duplicated genes is expected as any gene is on average represented by multiple alternatively spliced or alternatively assembled transcripts. A transcriptome-wide overview of the influence temperature, sex, and feeding treatment shows that sex is the major factor influencing the expression profile of individuals (Fig. 2). In general, females appear to be less diverse than males with respect to expression profiles. Additionally, temperature explains some diversity in expression profiles among samples, albeit its effect is much smaller than that of sex. To further analyze the effect of temperature – possibly dependent on sex and feeding treatment – on the expression of individual genes, statistical tests were performed for each assembled transcript and results were associated with the transcript’s cluster as a proxy for the gene. We first identified transcripts that are differentially expressed between the two temperatures. This was achieved by testing the null hypothesis that temperature has neither an overall influence on expression, nor is interacting with sex or feeding treatment, nor jointly with sex and feeding treatment, i.e. using the script null hypothesis *H*_0_ : *α*_3_ = *α*_5_ = *α*_7_ =  *α*_8_ = 0. Accordingly, the expression of 3489 transcripts in total was significantly affected by temperature. When testing the hypotheses *α*_3_ = 0, *α*_5_ = 0, *α*_7_ = 0 and *α*_8_ = 0 individually, we obtained 1280 transcripts that were exclusively affected by temperature (*α*_3_ ≠ 0), 134 transcripts with a sex-dependent influence of temperature (*α*_5_ ≠ 0), 18 transcripts with a feeding-dependent influence of temperature (*α*_7_ ≠ 0), and 5 transcripts where the effect of temperature depended on both food and sex (*α*_8_ ≠ 0) (Additional file 2: Data S1). Thus, the vast majority of transcripts was influenced by temperature independently of sex and feeding treatment. We here consider only those transcripts, which could be assigned to a NCBI gi number, which explains the slightly reduced numbers in the Additional file 2: Data S1.

The highest numbers of differentially expressed transcripts and clusters, respectively, were found to be affected by the factor sex, followed by temperature and the interaction between temperature and sex (Table 2; see also Additional file 2: Data S1). In contrast, the factor feeding regime and the remaining interactions caused differential gene expression in 5–29 transcripts/clusters only. In total, 13,570 transcripts and 6497 transcript clusters were significantly up- or down-regulated, showing annotation rates of 84.6% for the factor sex, 87.7% for temperature, and 88.5% for feeding regime.

**Table 2:** Number of differentially expressed transcripts and transcript clusters for different sources of variation

Source	Transcripts	Transcript clusters
Temperature	1242	1050
Sex	12,138	6363
Food	29	29
Temperature * Sex	126	111
Temperature * Food	17	16
Sex * Food	13	13
Temp. * Sex * Food	5	5

A transcript/cluster was considered significant if the false discovery rate, as determined by edgeR, was below 5%

**Fig. 3:**
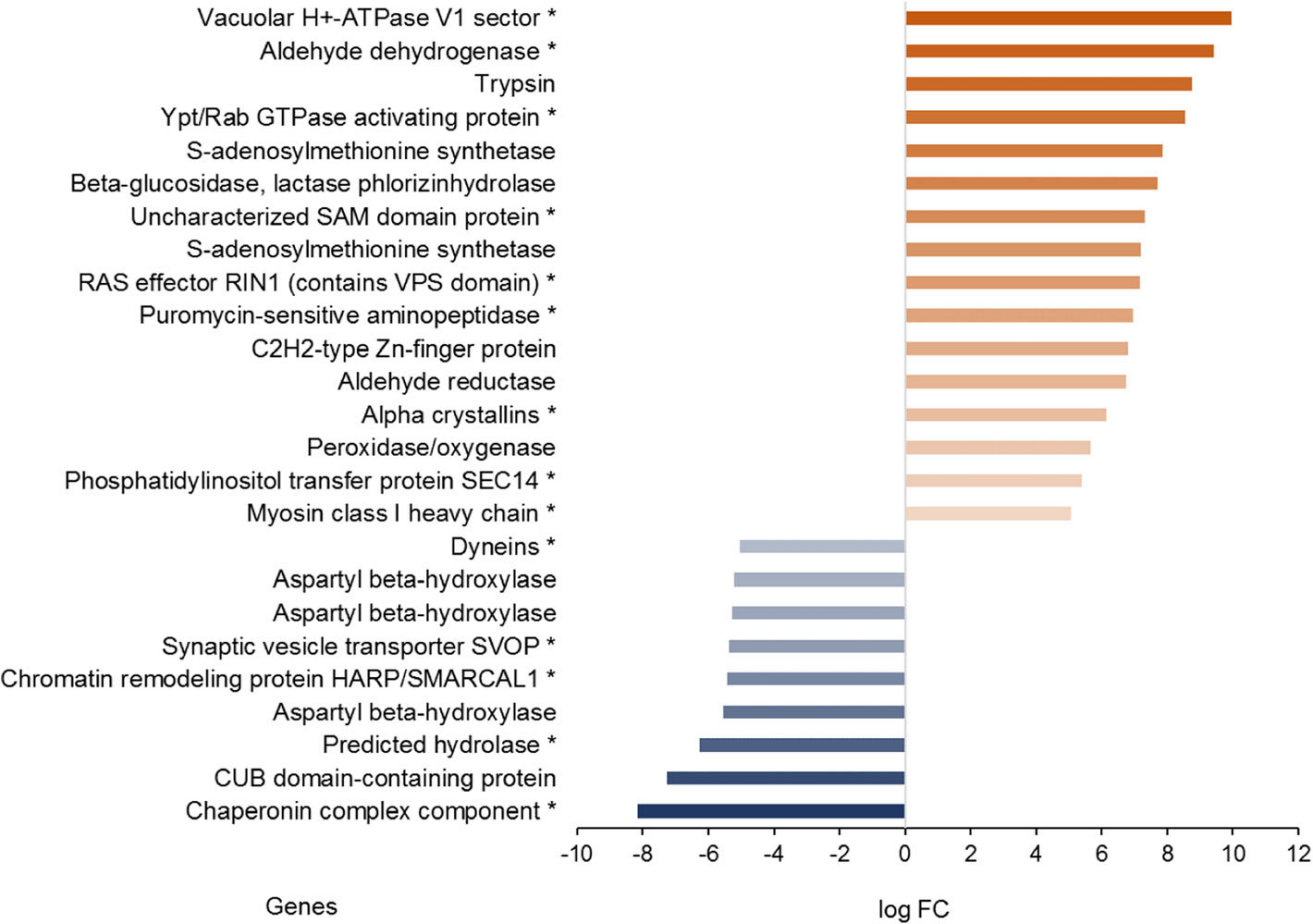
Overview of the genes being exclusively affected by the factor temperature. In these genes expression was independent of effects of sex and feeding treatment. Only genes with |logFC values| > 5 are shown. Red: up-regulation at the higher temperature; blue: down-regulation at the higher temperature. The darker the colour, the stronger the up- or down-regulation. Genes marked by * are private genes

Out of the 1089 annotated (out of 1242) transcripts that were affected by the factor temperature alone, 659 were down- and 430 up-regulated at the higher temperature. The genes most strongly down-regulated at the higher temperature were a chaperonin complex component and a CUB domain-containing protein, and the genes most strongly up-regulated at the higher temperature were vacuolar H+ ATPase V1 sector, aldehyde dehydrogenase, and trypsin (Fig. 3). Genes associated with oxidative defense (peroxidase/oxygenase) were also among the most strongly upregulated ones. The transcripts being down-regulated at the higher temperature were mainly associated with cellular processes and signaling (31.7%) and information storage and processing (28.7%; Table 3a). 20.0% of down-regulated transcripts were only poorly characterized. Regarding sub-functions, the highest numbers of down-regulated transcripts were associated with posttranslational modification, protein turnover, chaperones (12.3%), translation, ribosomal structure and biogenesis (7.9%), transcription (7.7%), and RNA processing and modification (7.4%). Transcripts up-regulated at the higher temperature were mainly associated with cellular processes and signaling (42.1%) followed by information storage and processing (25.1%) and metabolism (15.8%; Table 3b). 17.0% of up-regulated transcripts were poorly characterized. The most frequently up-regulated sub-functions included signal transduction mechanisms (22.1%), transcription (12.3%), posttranslational modification, protein turnover, chaperones (6.1%), and RNA processing and modification (6.1%). An enrichment analysis indicated 24 enriched functional categories at the higher compared to the lower temperature. Enriched functions mainly included metabolic and biosynthetic pathways, but most importantly also responses to oxidative stress (nitric oxide biosynthetic process; Table 4).

**Table 3:** Functional annotation of transcripts (a) down- or (b) up-regulated at the higher temperature according to molecular main and sub-role

Main role	Sub-role	N	%
A			
Cellular processes and signaling	Posttranslational modification, protein turnover, chaperones	81	12.3
	Signal transduction mechanisms	34	5.2
	Cytoskeleton	32	4.9
	Intracellular trafficking, secretion, and vesicular transport	27	4.1
	Cell cycle control, cell division, chromosome partitioning	17	2.6
	Defense mechanisms	6	0.9
	Cell wall/membrane/envelope biogenesis	4	0.6
	Extracellular structures	4	0.6
	Nuclear structure	3	0.5
	Cell motility	1	0.2
Information storage and processing	Translation, ribosomal structure and biogenesis	52	7.9
	Transcription	51	7.7
	RNA processing and modification	49	7.4
	Replication, recombination and repair	25	3.8
	Chromatin structure and dynamics	11	1.7
Metabolism	Energy production and conversion	36	5.5
	Carbohydrate transport and metabolism	29	4.4
	Amino acid transport and metabolism	21	3.2
	Lipid transport and metabolism	12	1.8
	Inorganic ion transport and metabolism	10	1.5
	Secondary metabolites biosynthesis, transport and catabolism	9	1.4
	Coenzyme transport and metabolism	7	1.1
	Nucleotide transport and metabolism	6	0.9
Poorly characterized	General function prediction only	72	10.9
	Function unknown	60	9.1
B			
Cellular processes and signaling	Signal transduction mechanisms	95	22.1
	Posttranslational modification, protein turnover, chaperones	26	6.0
	Cytoskeleton	24	5.6
	Intracellular trafficking, secretion, and vesicular transport	12	2.8
	Cell cycle control, cell division, chromosome partitioning	8	1.9
	Extracellular structures	7	1.6
	Cell motility	3	0.7
	Cell wall/membrane/envelope biogenesis	3	0.7
	Nuclear structure	2	0.5
	Defense mechanisms	1	0.2
Information storage and processing	Transcription	53	12.3
	RNA processing and modification	26	6.0
	Chromatin structure and dynamics	12	2.8
	Translation, ribosomal structure and biogenesis	9	2.1
	Replication, recombination and repair	8	1.9
Metabolism	Carbohydrate transport and metabolism	14	3.3
	Energy production and conversion	12	2.8
	Lipid transport and metabolism	12	2.8
	Amino acid transport and metabolism	9	2.1
	Inorganic ion transport and metabolism	7	1.6
	Secondary metabolites biosynthesis, transport and catabolism	7	1.6
	Nucleotide transport and metabolism	4	0.9
	Coenzyme transport and metabolism	3	0.7
Poorly characterized	General function prediction only	54	12.6
	Function unknown	19	4.4

The tables show the number of transcripts being exclusively affected by the factor temperature according to cellular processes and signaling, information storage and processing, metabolism and poorly characterized. Annotations are based on COG/KOG after Prophane

**Table 4:** Significantly enriched functional categories found to be a) down- or b) up-regulated at the higher temperature

No	Enriched functional category	*p*-value
A		
1	protein folding	1.70E-06
2	glycine decarboxylation via glycine cleavage system	5.60E-05
3	ATP synthesis coupled proton transport	3.30E-04
4	proteasome-mediated ubiquitin-dependent protein catabolic process	6.60E-04
5	glycolytic process	1.72E-03
6	SRP-dependent cotranslational protein	3.07E-03
7	protein catabolic process	3.64E-03
8	proteolysis involved in cellular protein	3.82E-03
9	histone peptidyl-prolyl isomerization	4.37E-03
10	DNA recombination	6.54E-03
11	protein refolding	8.71E-03
12	glycyl-tRNA aminoacylation	8.71E-03
13	protein N-linked glycosylation via aspar	1.30E-02
14	malate metabolic process	1.30E-02
15	mitochondrial pyruvate transport	1.74E-02
16	phenylalanyl-tRNA aminoacylation	2.17E-02
17	mitochondrial electron transport, NADH	2.17E-02
18	protein insertion into membrane	2.17E-02
19	Arp2/3 complex-mediated actin nucleation	3.44E-02
20	DNA replication	3.78E-02
B		
1	protein secretion	2.00E-03
2	nitric oxide biosynthetic process	8.10E-03
3	translational termination	1.50E-02
4	nucleosome assembly	1.51E-02

Only GO groups with FDR-corrected *p*-values < 0.05 were used

Regarding interactive effects, 110 annotated transcripts were significantly affected by temperature and sex. Of these, 49 were significantly more strongly down-regulated and 61 significantly more strongly up-regulated in females than in males at the higher temperature (Additional file 2: Data S1). The specific genes showing the largest sexual differences in their response to temperature were the RAS effector and lipid phosphate phosphatase (both down-regulated), and the allantoicase and CUB domain-containing protein (up-regulated). For combined effects of temperature and feeding treatment we found 14 annotated transcripts of which 9 were significantly more strongly down-regulated and 5 significantly more strongly up-regulated at the higher temperature under control conditions versus food restriction (Additional file 2: Data S1). The RNA-binding protein fusilli, trypsin, and molecular chaperones were more strongly down-regulated, whereas an extracellular matrix glycoprotein and a calcium transporting ATPase were more strongly up-regulated under control conditions versus food restriction. The interaction between sex and feeding regime revealed 12 annotated transcripts. Five of these were significantly more strongly down-regulated in females compared with males when being fed ad libitum, including F-box proteins and prohibitins and stomatins, while 7 were significantly more strongly up-regulated. Finally, 5 annotated transcripts were affected by the 3-way interaction. Three were down-regulated (thioredoxin binding protein, trypsin, myosin) and 2 up-regulated (RAS effector RIN1, transcription factor zerknullt) at the higher temperature in females compared to males when being fed ad libitum.

Regarding sex differences in gene expression, out of 10,265 annotated differentially expressed transcripts 4853 were down- and 5412 up-regulated in females relative to males. The specific genes most strongly down-regulated in females were trypsin and a nuclear pore complex component, and the genes most strongly up-regulated in females were a lipoprotein and DNA polymerase alpha (Additional file 3: Figure S1). The transcripts being down-regulated in females were mainly related to metabolism (36.7%) and cellular processes and signaling (32.1%), while those being up-regulated were mainly associated with information storage and processing (36.3%) and cellular processes and signaling (32.2%; Additional file 3: Figure S2). We found 85 enriched functional categories in females compared with males, the majority of which was associated with metabolism (Additional file 4: Data S2). Furthermore, up-regulated enriched functional categories in females included stress responses such as DNA repair, heme oxidation, and protein refolding. See below for potential effects of dosage compensation on sexual differences in expression patterns.

Only 26 annotated transcripts were differentially expressed among feeding treatments, of which 14 were down-regulated and 12 up-regulated under control conditions relative to food restriction. Genes most strongly up-regulated under control conditions were phosphatidylinositol transfer protein SEC14 and prohibitins and stomatins, and genes most strongly down-regulated were pyruvate kinase and the thiamine pyrophosphate-requiring enzyme (Additional file 3: Figure S1). Seven of the down-regulated transcripts were related to cellular processes and signaling, three to metabolism, and two to information storage and processing. We found five up-regulated transcripts related to metabolism, three to cellular processes and signaling, and two to information storage and processing (Additional file 3: Figure S2). We found four enriched functional categories affected by feeding treatment (Additional file 4: Data S2).

## Discussion

### Variation in life history and physiological traits

Our data show that the adult treatments employed were effective in changing phenotypic traits, as evidenced by significant effects on thorax mass, abdomen mass, thorax-abdomen ratio, fat content, and lysozyme activity. Under cooler conditions, butterflies had heavier thoraces but lighter abdomen and consequently a higher thorax-abdomen ratio, and food-restricted animals also had a higher thorax-abdomen ratio. These differences probably reflect variation in energy expenditure and acquisition. Higher temperatures and food access enable higher levels of food acquisition, shifting thorax-abdomen ratios towards lower values. This interpretation is in line with the results on fat content, being or tending to be higher at the warmer temperature and unrestricted access to food [6, 39]. Lysozyme activity was reduced at the higher temperature and under food restriction, corroborating earlier findings indicating detrimental effects of such conditions on immune competence [40, 41]. The temperatures used here also induce substantial variation in other traits in *B. anynana*, including cold and heat stress resistance, egg size, egg composition, fecundity, and reproductive output [6, 8, 36, 42–44].

As expected, *B. anynana* showed also pronounced sexual differences. Higher female masses are frequently found in insects, which is likely caused by fecundity selection [39, 41, 45]. The males’ higher relative fat content probably reflects their higher energy demand to sustain high flight activity e.g. during mate location [6, 46]. Concomitantly, males also show a higher thorax-abdomen ratio [47, 48]. Regarding physiological traits, the higher lysozyme activity in females may reflect an increased investment into immune function and thereby longevity to maximize reproductive output [49], while their higher ADH activity (cf. [34] may reflect higher metabolic rates which may in turn increase egg production [50]. Three sex by temperature interactions were significant, indicating that females were able to benefit more strongly from the higher temperature and thus exemplifying sex-specific responses to acclimation temperatures. Other examples of sex-specific temperature effects in butterflies include heat shock protein expression and longevity [39, 41].

### Effects of acclimation temperature on gene expression

The vast majority of assembled transcript clusters mapped to genes of other insects and especially other Lepidoptera, indicating that we managed to de novo assemble a high-quality transcriptome of the butterfly *B. anynana*. Although we have deliberately chosen to focus on short-term adult acclimation, acclimation temperature had a significant impact on gene expression (hypothesis 1). The non-stressful adult temperatures used here do cause striking variation in phenotype, and based on the expression patterns found we conclude that those shifts are induced by changes in a large number of pathways.

Overall, fewer genes were up- than down-regulated at the higher temperature, the former including pathways associated with signal transduction, transcription, posttranslational modification, protein turnover, chaperones, RNA processing and modification, cytoskeleton, and lipid metabolism. For all these processes also down-regulation at the higher temperature was found. Thus in contrast to hypothesis 1, we could not find strong evidence for an overall upregulation of genes related to metabolism at the higher temperature. Nevertheless, several genes with particular high logFC values were related to metabolism, such as ATPase, aldehyde dehydrogenase, trypsin, S-adenosylmethionine synthetase, and beta-glucosidase. These findings may reflect the fact that, in ectotherms, metabolism and enzymatic activity generally increase with increasing temperature [51, 52]. Metabolic activity may also increase at (stressfully) low temperatures [53] or may remain unaffected by temperature variation [54, 55], which may explain the rather high level of down-regulation at the higher temperature found here.

Our principal aim though was to identify candidate pathways that may confer higher heat tolerance after exposure to non-stressful warm temperatures. Interestingly, we indeed found a strong upregulation of genes related to the oxidative stress response at the higher temperature as predicted, including for instance peroxidase/oxygenase (Fig. 3), the Cytochrome P450 superfamily (Additional file 2: Data S1; cf. [56]), heme binding, and oxidoreductase activity (Additional file 2: Data S1). Also, the functional category ‘nitric oxide biosynthetic process’ was overrepresented at the higher temperature. Higher temperatures elevate the production of reactive oxygen species as a by-product of the aerobic metabolism, which may cause oxidative damage [57, 58]. A concomitant up-regulation of antioxidant enzymes may in turn alleviate the resulting oxidative stress, and has therefore been implied to play a crucial role for heat stress resistance in insects [59–62]. Antioxidant enzymes, in particular superoxide dismutase, have also been implied to play an important role in longevity and starvation resistance in *B. anynana* and other animals [63–65]. Furthermore, responses to oxidative stress may mediate the trade-off between reproduction and longevity, at least under stressful thermal conditions [66]. Thus, upregulation of antioxidant enzymes comprises a straightforward candidate mechanism that may underlie increased heat tolerance after acclimation to warmer temperatures, helping to safeguard homeostasis if subsequently exposed to stressful conditions.

In contrast, we found no support for a meaningful involvement of the heat shock response in acclimating to warmer temperatures. The heat shock response is basically an emergency reaction under acute stress [37]. Hence, we assume that the non-stressful acclimation temperatures used here were not high enough to induce the heat stress response (cf. [67]). Interestingly, similar temperatures did up-regulate heat-shock proteins in a temperate-zone Copper butterfly [68]. This suggests that the tropical butterfly investigated here, in contrast to the aforementioned temperature-zone species, did not experience 27 °C as being stressful and that consequently higher temperatures are needed for up-regulation [53, 67, 69].

### Effects of acclimation temperature in interaction with other factors on gene expression

At least some transcripts were involved in the interaction between feeding treatment and temperature, in line with our second hypothesis. Thus, we indeed found evidence that inadequate nutrition may interfere with plastic responses, as evidenced for instance by a stronger up-regulation of molecular chaperones at the lower temperature in the feeding as compared with the food-restricted group. Also the fusilli protein, which is important in embryogenesis and other developmental processes [70], was significantly more strongly down-regulated at 27 °C in the feeding as compared with the food-restricted group. This may in turn impact food intake, ensuring growth and eventually maturation [10].

As expected, sexes responded in part differently to temperature, evidenced by significant sex by temperature interactions for expression patterns. This interaction affected the expression of 111 annotated genes. Examples include genes associated with energy production and lipid transport (lipid phosphate phosphatase, Di-hydrolipoamide acetyltransferase). These were more strongly down-regulated in females than in males at the higher temperature. While sex specific responses to temperature clearly do occur, many traits including temperature stress resistance show similar patterns in both sexes (e.g. [10, 40, 41, 67]), which may explain the relatively low incidence of interactive effects.

Only five transcripts were affected by the 3-way interaction. We found a strong down-regulation of the thioredoxin binding protein, which plays an important role in redox homeostasis by increasing reactive oxygen species and oxidative stress [71], at the higher temperature in females compared with males when being fed ad libitum. This suggests that females, under normal feeding conditions at 27 °C, were less exposed to oxidative stress than the other groups. Furthermore, the RAS effector RIN1 was up-regulated at the higher temperature in females compared with males when being fed ad libitum, which plays a pivotal role in activating various cellular processes [72]. Thus, females activate cellular processes under favourable conditions.

### Effects of feeding treatment and sex on gene expression

The fact that only a small number of transcripts was differentially expressed among feeding treatments suggests that butterflies could largely compensate for the temporary lack of food (see also [73]). However, at least some functions seem to have been compromised by limited food access, evidenced by changes in thorax-abdomen ratio, lysozyme activity, and fat content (see above). Accordingly, at least a few metabolic functions such as carbohydrate and amino acid transport and metabolism were down-regulated under food restriction.

We found pronounced sexual differences in expression patterns as expected, being at least partly related to reproduction. For example, the genes most strongly up-regulated in females as opposed to males were lipoproteins, being important for egg production [74, 75]. Furthermore, metabolism (e.g. trypsin, enolase, fatty acid desaturase) and innate immunity (e.g. serpin) appeared to be down-regulated in females compared with males. Whether the overall down-regulation of genes related to innate immunity and metabolic pathways indicates a trade-off between investment into maintenance versus reproduction (cf. [66, 76]), requires further investigation. To what extant sex-specific differences in gene expression are affected by (a lack of) dosage compensation in butterflies, in which females are the heterogametic sex, is currently unknown [77–80]. Only very few genes were differentially expressed between the sexes in interaction with feeding regime, suggesting that food stress had similar effects in both sexes.

## Conclusions

Based on a high-quality transcriptome, our study revealed that, as expected, sex had overall the largest impact on expression patterns. At the higher temperature, several antioxidant markers were up-regulated, which may play an important role in the acclamatory processes subsequently providing increased heat tolerance. In contrast, the heat shock response did not seem to be involved in the acclamatory response. We thus believe that the role of anti-oxidative responses in increasing heat tolerance may have been hitherto underestimated. Their upregulation may provide a crucial mechanism to prepare organisms for later more stressful conditions. Such conditioning may arise from higher basal levels of anti-oxidant enzymes or a quicker response to deteriorating conditions as the respective ‘machinery’ has been already turned on. While the important role of antioxidant enzymes in alleviating detrimental effects of oxidative stress is firmly established [58, 81], we believe that its potentially crucial role for mediating increased stress tolerance, even under non-stressful conditions, deserves more attention (e.g. [59, 62, 65, 82, 83]). Additionally, we provide molecular evidence that plastic responses may be compromised by inadequate nutrition. Our study though shows that even a few days at different temperatures in the adult stage affect expression patterns and phenotypes including thermal stress resistance [10]. Under natural conditions, the phenotypic changes induced (namely increased heat tolerance) are likely to sustain longer lifespans, higher levels of activity, and a higher reproductive output under stress (cf. [12, 13]).

## Methods

### Study organism

*Bicyclus anynana*, a tropical fruit-feeding butterfly, is distributed from southern Africa to Ethiopia [84]. As an adaptation to alternate wet-dry seasonal environments and the associated changes in resting background and predation, this species exhibits striking phenotypic plasticity (two seasonal morphs; [7]). Reproduction is confined to the favorable wet season during which oviposition plants are abundantly available [35, 85]. In 1988, a laboratory stock population was established at Leiden University, the Netherlands, from over 80 gravid females collected at a single locality in Malawi. Several hundred adults are reared in each generation, maintaining high levels of heterozygosity at neutral loci [86]. From the Leiden stock population, a laboratory population was established at Greifswald University, Germany, in 2007 from which all animals for this experiment originated.

### Experimental design

To start this experiment, freshly eclosed virgin males and females (50 each) from the stock population were mated. Single-mated females were afterwards kept individually in 1 L translucent plastic containers for oviposition to create full-sib families. They were provided with cuttings of maize as oviposition substrate and moist banana for adult feeding on a daily basis. All females and their offspring were kept in a common environment inducing wet season phenotypes, i.e. at 23 °C, 70% relative humidity, and a photoperiod of L12:D12. The eggs laid by individual females were removed from the containers and transferred to petri-dishes. After hatching, larvae were transferred individually to small plastic pots (125 ml) lined with moist paper tissue and were fed on cuttings of young maize plants being replaced every other day. Following adult eclosion, butterflies were randomly allocated to one out of four treatment groups, differing in temperature (19 °C or 27 °C) and feeding treatment, using a split-family design (*n* = 12 families, *n* = 494 individuals). Butterflies were, based on pilot experiments, either fed with moist banana ad libitum (control) or had access to banana for only 30 min/48 h, resulting in four treatment groups: (1) 19 °C control (*n* = 122), (2) 19 °C food-restricted (*n* = 127), (3) 27 °C control (*n* = 123), (4) 27 °C food-restricted (*n* = 122). The above temperatures were chosen due to their ecological relevance and because they were known to induce large differences in thermal tolerance and other traits within a few days [8, 10]. On day 8 of adult life, all butterflies were shock-frozen in liquid nitrogen and stored at − 80 °C for later analyses. Food-restricted butterflies were not fed for ca. 44 h before being frozen to maximize effects on phenotypes and expression patterns.

### Life-history traits and physiological measurements

As proxies of condition, we scored adult body mass, thorax mass, abdomen mass, thorax-abdomen ratio, fat content, lysozyme activity, alcohol dehydrogenase (ADH) activity, and protein content for all individuals. Lysozyme activity was included as an indicator of investment into immune function [49], while ADH activity may reflect metabolic rates [34, 50]. For subsequent measurements, head, legs, and wings were removed, and abdomen and thorax separated. All masses were weighed as fresh mass to the nearest 0.01 mg (Sartorius microscale LE225D, Sartorius AG, Goettingen, Germany). Butterfly abdomens were used for measuring relative fat content, except for individuals used for RNA extractions (see below). Fat content was determined as difference in abdomen dry mass before and after two fat extractions in percent. Therefore, abdomens were dried to constant mass at 60 °C for 48 h. For fat extractions, 0.4 ml of dichloromethane (CH_2_Cl_2_) per abdomen was applied for 24 h (cf. [87]).

For the determination of lysozyme activity, ADH activity, and protein content, the thoraces were used. Each thorax was homogenized in 100 μl phosphate-buffered saline (PBS; 11.9 mM Na_2_HPO_4_ * 2 H_2_O, 137 mM NaCl, 2.7 mM KCl, pH 8.0) and centrifuged at 4 °C for 20 min (14,000 rpm). Lysozyme activity was determined following [88]. The wells of a 96-well plate were loaded with 20 μl supernatant plus 80 μl *Micrococcus luteus* solution (3 mg/ml in PBS) or blanks containing 20 μl PBS plus 80 μl *M. luteus* solution. The optical density was measured at 490 nm and 30 °C for 5 h (microplate reader BioTekELx 808, Bad Friedrichshall, Germany). For the determination of ADH activity, wells were loaded with 10 μl supernatant plus 190 μl reaction solution (30 mM isopropanol [23 μl/10 ml TRIS HCl], 3 mM NAD+ [0.0199 g/10 ml TRIS HCl], 0.15 M TRIS HCl buffer pH 8.5) or blank samples (with 10 μl PBS plus 190 μl reaction solution). ADH activity was measured as change in the optical density at 340 nm and 30 °C for 10 min (15 s steps; BioTekELx 808). Both activity measures were calculated by subtracting the final from the initial value, and by additionally correcting for the mean of the blank values. Total protein content was quantified using the BioRad protein assay based on the Bradford method [89]. Therefore, 1 μl of the supernatant was diluted in 160 μl aqua dest. After adding 40 μl BioRad solution and 10 min of incubation, the absorbance was read at 595 nm and 30 °C (BioTekELx 808). Four replicates were measured per individual. A standard curve was constructed with albumine bovine serum in cacodylate buffer, using a concentration series (0–2 mg/ml).

Data on life-history and physiological traits were analyzed using analyses of variance (ANOVAs) with adult temperature, adult feeding treatment, and sex as fixed effects and family as random factor. To test for normality and variance homogeneity, we used the Kolmogorov-Smirnov and the Levene test, respectively. If necessary, data were transformed as appropriate. Pair-wise comparisons were performed employing Tukey’s HSD. Minimum adequate models were constructed by sequentially removing non-significant interaction terms. The above statistical tests were performed using Statistica (8.0). Throughout the text, estimates are stated as mean ± standard error.

### RNA isolation and purification

Whole abdomens were used for RNA extraction (*n* = 80), as the thoraces were used for the above analyses. We selected 10 butterfly families (i.e. the offspring of 10 single-mated females). RNA was extracted from one individual per family and treatment group (i.e. per 2 temperatures * 2 feeding treatments * 2 sexes, resulting in 8 individuals per family). Total sample size was 77 instead of 80 individuals (8 * 10 families), as RNA quality was insufficient in 3 individuals from 27 °C. RNA isolation was carried out using TRIZOL (Invitrogen) according to the manufacturer’s instructions, using one ml of TRIZOL per abdomen. For RNA purification, the RNeasy Mini kit (Qiagen 74,106) and the RNAase free DNAase kit (Qiagen 792,454) were used. The RNA was eluted in 100 μl and stored at − 80 °C for later analysis (for a detailed description see Additional file 5).

### Sequencing, transcriptome assembly, and expression analysis

After RNA extraction, mRNA of eukaryotes was enriched by using oligo(dT) magnetic beads. By adding fragmentation buffer, the mRNA was interrupted to short fragments (about 200 bp). Then, the first strand of cDNA was synthesized by a random hexamer-primer using the mRNA fragments as templates. Buffer, dNTPs, RNase, and DNA polymerase were added to synthesize the second strand. The double strand cDNA was purified with the QiaQuick PCR extraction kit and washed with EB buffer for end repair and single nucleotide A (adenine) addition. Finally, sequencing adaptors were ligated to the fragments. The required fragments were purified by agarose gel electrophoresis and enriched by PCR amplification.

Each sample was sequenced as a separate paired end library on a HiSeq2000 Illumina platform by BGI Hong Kong Co. Ltd., with 9 to 10 samples per lane. In total we obtained 2,066,491,822 unstranded paired-end reads (100 bp read length), with 8–14 million reads per individual. As its usefulness is under debate, no filtering and trimming was applied (except for adaptors) [90]. Additionally, using quality trimming in the Trinity assembler resulted in only 0.5% fewer contigs, and was therefore deemed unnecessary. Given that a reference genome of *B. anynana* is not available, we performed a de novo assembly. We used and compared two popular software packages, SOAP denovo-trans (version 1.02; [22]) and Trinity (version r2013-02-25; [91]). We used two quality measures to choose between both assemblies and to reduce the number of contigs to meaningful transcripts. First, we aligned the transcripts against the proteins of the model organism *Drosophila melanogaster* as quality criterion (Additional file 6: Table S2). The analyses indicated that using SOAP 1574 proteins aligned with at least 80% coverage, whereas in the case of Trinity 3694 proteins did so. Second, the most likely coding regions were extracted from both assemblies. Accordingly, a total of 25,016 SOAP and 66,712 Trinity transcripts that correspond to likely coding regions were obtained. Based on both quality measures Trinity produced a better assembly, such that the SOAP assembly was discarded.

For the Trinity assembly, transcript abundances were computed with eXpress (version 1.3.1; 10.1038/nmeth.2251). Reads were mapped to the transcript assembly with Bowtie 2 [92]. Default values of eXpress and Bowtie 2 were used except for parameter B (additional number of EM iterations) which was set to 20. Abundance levels are reported as reads per kilobase of transcript per million mapped reads (RPKM; [93]). To identify differential expression of assembled transcripts, eXpress [94] was used to estimate the relative expression count of each assembled transcript for each individual. ‘Effective counts’, comprising the value recommended for input to count-based differential expression tools (https://pachterlab.github.io/eXpress/manual.html), were rounded to the nearest nonnegative integer. For 30 out of the 66,712 assembled transcripts this count could not be computed by eXpress such that they were subsequently ignored. The resulting 66,682 × 77 matrix was statistically analyzed with edgeR 3.16.5 [95].

First, samples were visualized in two dimensions using multidimensional scaling (function plotMDS of edgeR), which revealed that two samples (one male, one female) were likely wrongly labeled (Additional file 3: Figure S3). These two samples were subsequently removed, leaving 75 samples. Very lowly expressed transcripts were not considered in the differential expression analysis: we ignored transcripts that did not have more than 50 reads in at least 5 samples. Libraries were normalized for statistical comparison using the standard trimmed mean of M-values of edgeR (calcNormFactors). The count data was assumed to follow a negative binomial distribution. A full-factorial generalized linear model was used to model the expression counts, with the binary factors temperature *t* (19 °C or 27 °C), sex *s* (male or female), and feeding treatment *f* (ad libitum or food stress) including all possible interactions.

$$ \ln {\mu}_{g,j}={\alpha}_1+{\alpha}_2{s}_j+{\alpha}_3{t}_j+{\alpha}_4{f}_j+{\alpha}_5{s}_j{t}_j+{\alpha}_6{s}_j{f}_j+{\alpha}_7{t}_j{f}_j+{\alpha}_8{s}_j{t}_j{f}_j $$

Here, *μ*_*g*, *j*_ is the mean expression of transcript *g* in sample *j* and the variables *s*_*j*_, *t*_*j*_ and *f*_*j*_ assume either the value 0 or 1. For an alternative approach with separate analyses for each sex see Additional file 7. *P*-values were adjusted for multiple testing with the Benjamini-Hochberg method [96]. Afterwards, we explored the effects of temperature on gene expression, which was the principle aim of this study, in more detail. Therefore, we compiled for each null hypothesis outlined below (see Results) a list of significant transcripts. Significance was determined using a false discovery rate (FDR) threshold of 5%.

The 66,712 assembled transcripts were aligned against the hexapoda section of the non-redundant protein database (February 2015) using blastx version 2.2.26 [97]. 96% of the assembled transcripts (64,047) had a significant hit at an e-value threshold of 10^− 5^, indicating that a large fraction of assembled transcripts contained protein-coding sequences. We further applied BUSCO version 3 [98] to assess the completeness of the set of assembled transcripts that were likely to contain coding sequences (see Results). As several transcripts may map to the same gene (e.g. alternative splicing, incomplete assembly), they were assigned to clusters when having a hit to the same target protein (single-linkage clustering). For clustering, only hits with (1) an e-value below 10^− 5^ that (2) belong to the top ten hits of a transcript and for which (3) the e-value is not larger than a thousand times the smallest e-value for that transcript were considered. These filters were introduced to prevent too broad clusters resulting from domains that are shared between many proteins. Each of the resulting 10,696 clusters thus likely represents assembled transcripts from the same gene or group of homologs.

The assembled transcript clusters were annotated against cluster of orthologous groups (COG) and eukaryotic orthologous groups (KOG; RPS-BLAST algorithms) using the program Prophane [99] and NCBI gi numbers (Additional file 2: Data S1). The COG/KOG system distinguishes among three protein main roles and 23 sub-roles. Additionally, Blast2GO (www.blast2go.com [100]) was used to assign transcript clusters to gene ontology (GO) terms (http://geneontology.org; Additional file 8: Data S3). As both annotations gave very similar results, we only present the results based on the COG/KOG system here (for results based on the GO system see Additional file 9). We finally performed enrichment analyses based on GO terms. GO IDs were retrieved using Interproscan v5.27–66.0. For the detection of significantly enriched GO terms, we used a Fishers exact test as implemented in the R package TopGo [101]. The complete contig set was taken as reference, with the sets of treatment-specific expressed genes comprising the test sets.

## Additional files

Additional file1: **Table S1.** Distribution of transcript clusters across taxa. (DOCX 13 kb)

Additional file 2: **Data S1.** This data file contains all original data from the differential expression analysis based on cluster of orthologous groups and eukaryotic orthologous groups. (XLSX 1045 kb)

Additional file 3: **Figures S1-S3. Figure S1.** Gives an overview of the specific genes most strongly affected by the factors sex and feeding regime. **Figure S2** depicts the functional annotation of transcripts being down- or up-regulated in females relative to males, and being down- or up-regulated under food ad libitum relative to food restriction. **Figure S3.** shows a multidimensional scaling plot for detecting outliers. Two outlier samples were assumed to have a mislabelled sex and were therefore excluded from further analyses. (DOCX 875 kb)

Additional file 4: **Data S2.** This data file contains all original data from the enrichment analyses. (XLSX 17 kb)

Additional file 5: RNA isolation and purification are described here in detail. (DOCX 13 kb)

Additional file 6: **Table S2.** The table shows the distribution of protein coverage for the Trinity and SOAP assemblies of the *Bicyclus anynana* transcriptome. Trinity produced a better assembly such that only Trinity was used in further analyses, whereas the SOAP assembly was discarded. (DOCX 13 kb)

Additional file 7: Here a justification is given for analysing males and females in a joint analysis. (DOCX 14 kb)

Additional file 8: **Data S3.** This data file contains all original data from the differential expression analysis based on gene ontology terms. (XLSX 2402 kb)

Additional file 9: This file gives an overview of the results based on gene ontology terms. The assembled transcript clusters were annotated against both, cluster of orthologous groups/eukaryotic orthologous groups (COG/KOG) as well as gene ontology terms. As both annotations gave very similar results, we only present the results based on the COG/KOG system in the main text. (DOCX 461 kb)
