# Supplementary material for: Effects of adult temperature on gene expression in a butterfly: identifying pathways associated with thermal acclimation
Source: BMC Evol Biol. 2019 Jan 23;19:32. doi: 10.1186/s12862-019-1362-y (PMC6345059; doi:10.1186/s12862-019-1362-y)
Supplement: Supplementary file 1 — Table S1. Distribution of transcript clusters across taxa. (DOCX 13 kb) [file 12862_2019_1362_MOESM1_ESM.docx]

**Additional file1: Table S1**

**Distribution of transcript clusters across taxa**

Distribution of the 10,696 clusters of assembled transcripts across taxa. The table is based on an alignment against the hexapoda section of the non-redundant NCBI protein database using blastx version 2.2.26.

| **Taxon** | **Transcripts** |
| --- | --- |
| Lepidoptera | 9995 |
| Hymenoptera | 215 |
| Diptera | 200 |
| Coleoptera | 117 |
| Hemiptera | 100 |
| Isoptera | 47 |
| Phtiraptera | 12 |
| Gammaproteobacteria | 4 |
| Blattodeo | 2 |
| Neuroptera | 1 |
| Orthoptera | 1 |
| Thysanoptera | 1 |
| Trichoptera | 1 |
| Primates | 1 |
