# Supplementary material for: Effects of adult temperature on gene expression in a butterfly: identifying pathways associated with thermal acclimation
Source: BMC Evol Biol. 2019 Jan 23;19:32. doi: 10.1186/s12862-019-1362-y (PMC6345059; doi:10.1186/s12862-019-1362-y)
Supplement: Supplementary file 3 — Figures S1-S3. Figure S1. Gives an overview of the specific genes most strongly affected by the factors sex and feeding regime. Figure S2 depicts the functional annotation of transcripts being down- or up-regulated in females relative to males, and being down- or up-regulated under food ad libitum relative to food restriction. Figure S3. shows a multidimensional scaling plot for detecting outliers. Two outlier samples were assumed to have a mislabelled sex and were therefore excluded from further analyses. (DOCX 875 kb) [file 12862_2019_1362_MOESM3_ESM.docx]

**Additional file 3: Figures S1-S3**


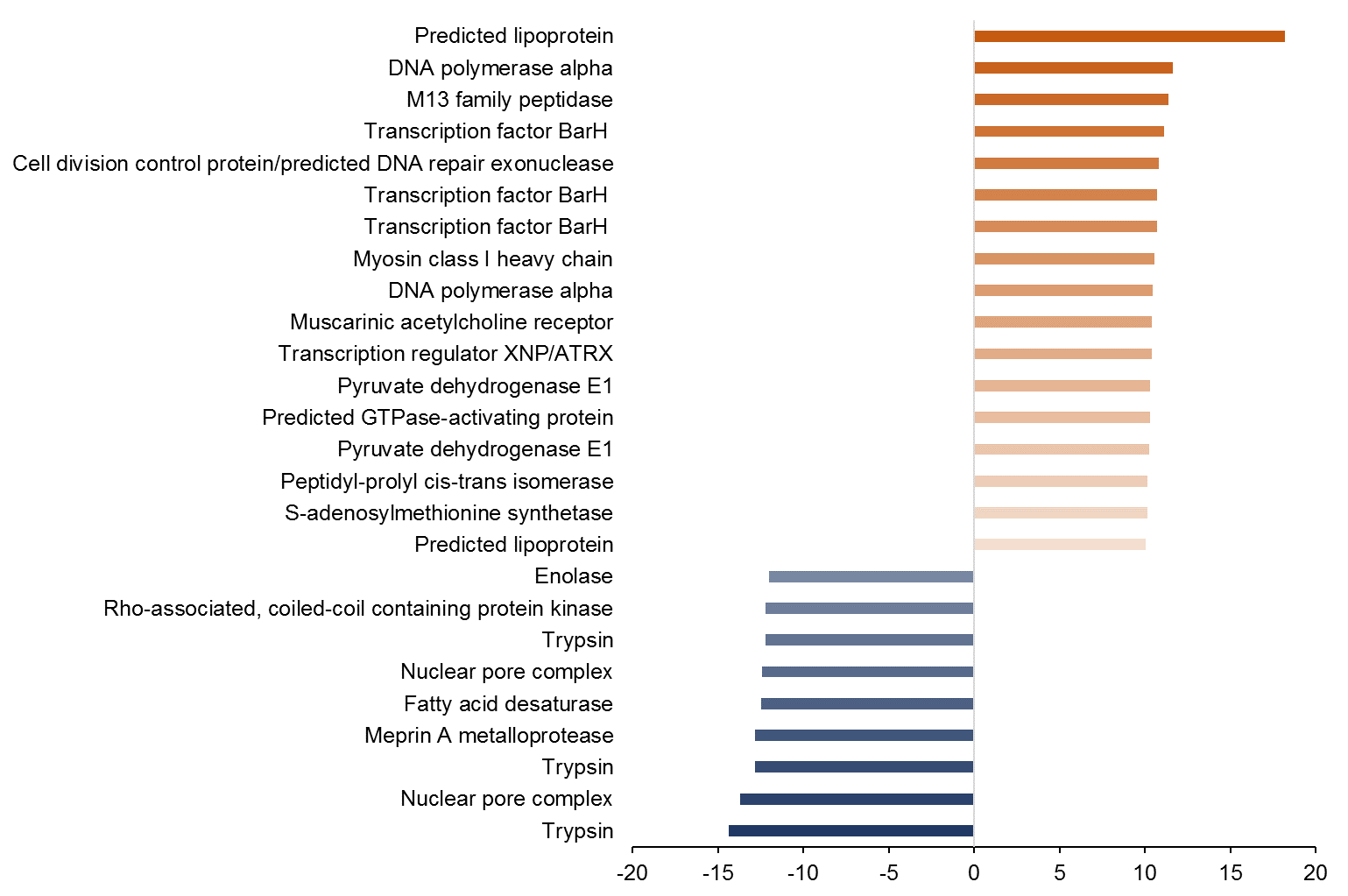


Figure S1a


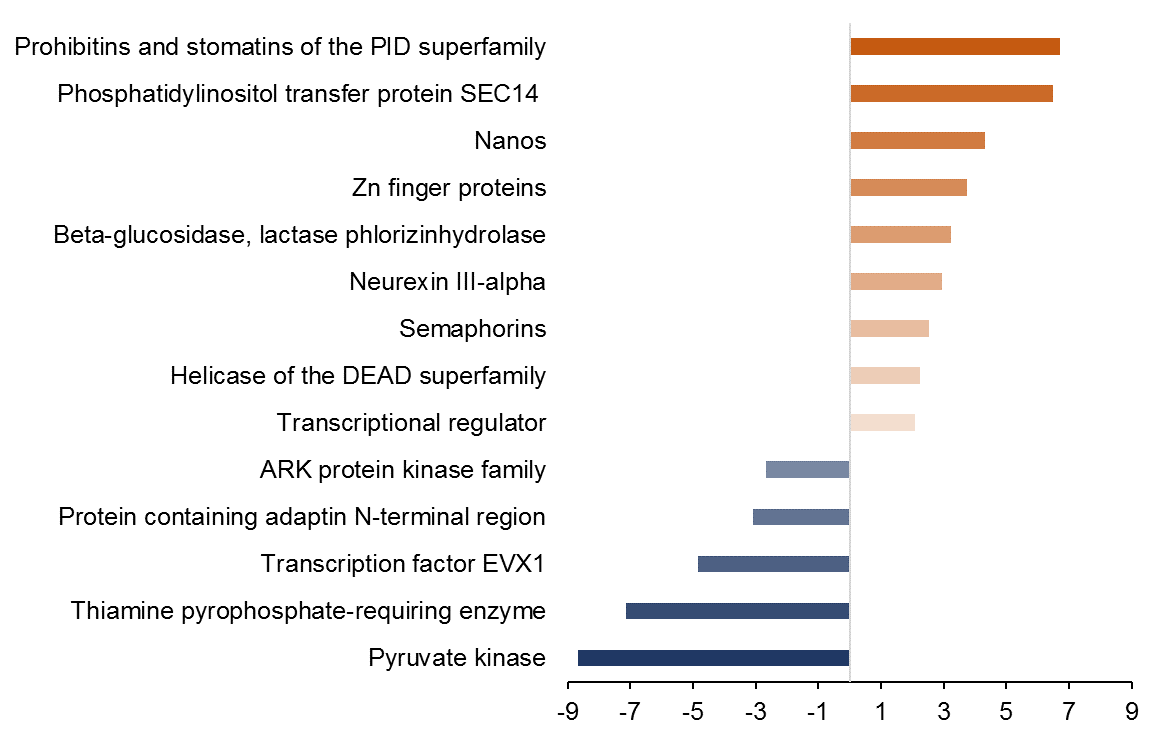


Figure S1b

**Figure S1:** Overview of genes in which expression was most strongly affected by the factors sex (a) and feeding regime (b). In (a) genes with logFC values > 10 and < -12 and in (b) > 6 and < -8 were included. Blue: down-regulation in females, and under food *ad libitum*; red: up-regulation in females, and under food *ad libitum*. The darker the colour the stronger the up- or down-regulation.


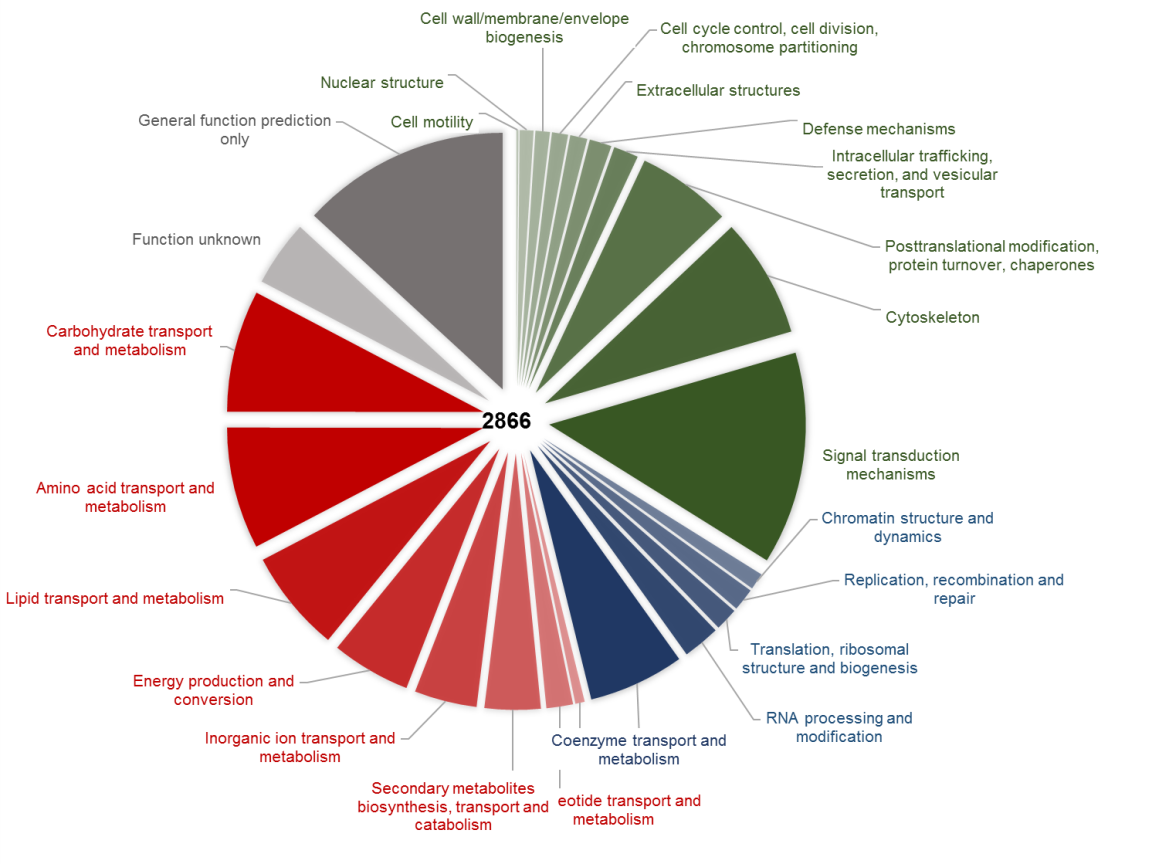


Figure S2a


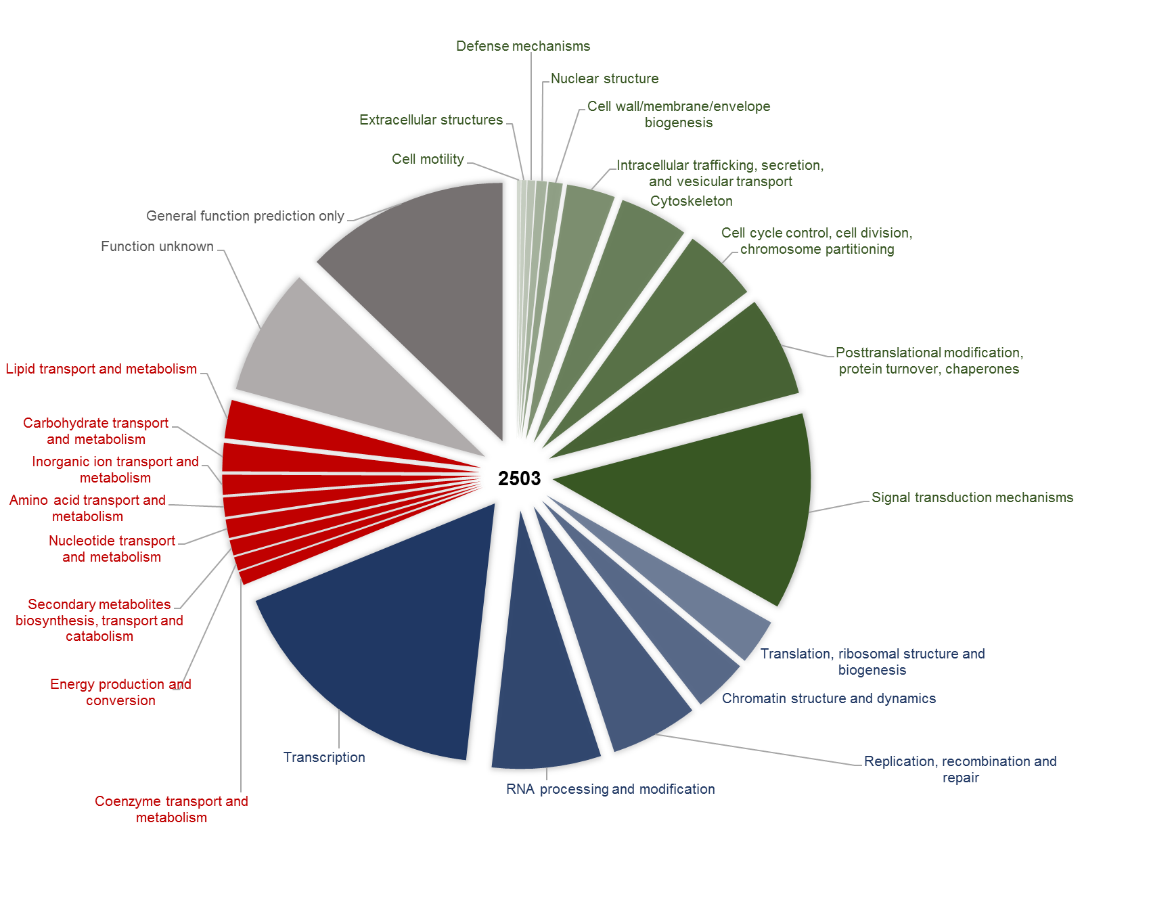


Figure S2b


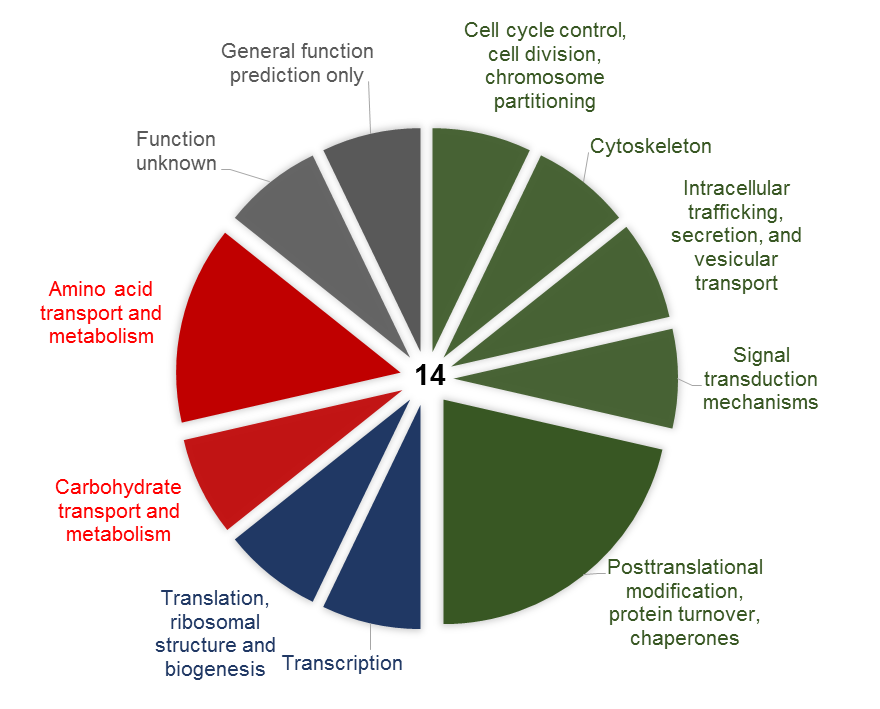


Figure S2c


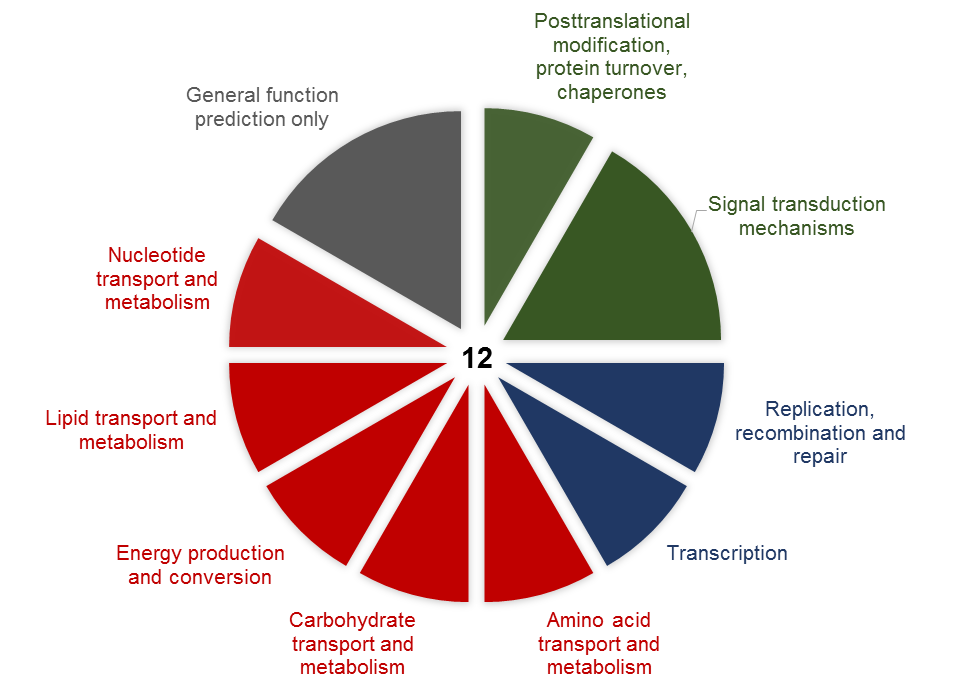


Figure S2d

**Figure S2:** Functional annotation of transcripts (COG / KOG after Prophane, [www.prophane.de](http://www.prophane.de/)) being down- (a) or up-regulated (b) in females relative to males, and being down- (c) or up-regulated (d) under food *ad libitum* relative to food restriction. Green: cellular processes and signalling; blue: information storage and processing; red: metabolism; grey: poorly characterized. Numbers represent the numbers of down- and up-regulated transcripts.


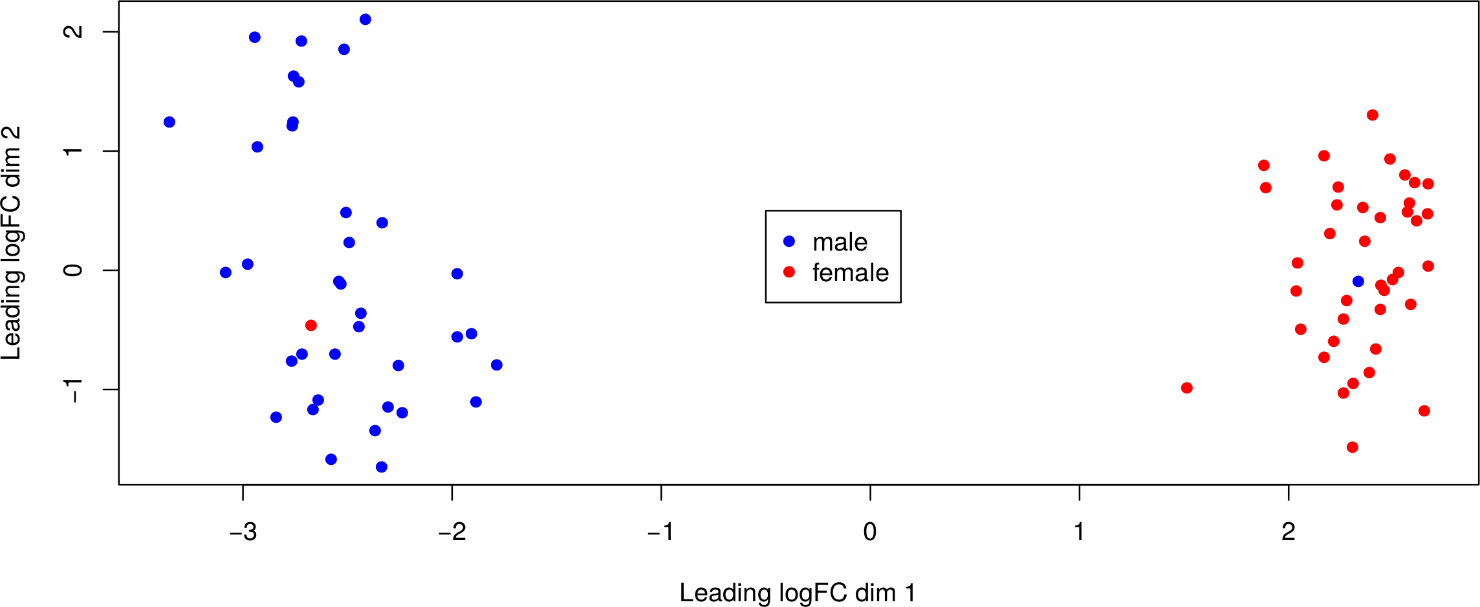


**Figure S3:** Multidimensional Scaling (MDS) plot of all 77 samples for detecting outliers. Points are placed proximal if their expression profiles are similar. The clear clustering of samples according to sex suggests that sex has a major influence on gene expression. Additionally, the two outlier samples (red dot at left and blue dot at right) were assumed to have a mislabelled sex and were therefore excluded from further analyses.
