# Supplementary material for: Effects of adult temperature on gene expression in a butterfly: identifying pathways associated with thermal acclimation
Source: BMC Evol Biol. 2019 Jan 23;19:32. doi: 10.1186/s12862-019-1362-y (PMC6345059; doi:10.1186/s12862-019-1362-y)
Supplement: Supplementary file 5 — RNA isolation and purification are described here in detail. (DOCX 13 kb) [file 12862_2019_1362_MOESM5_ESM.docx]

**Additional file 5**

**RNA isolation and purification**

One ml of TRIZOL was added to each abdomen, which were thereafter disrupted using a tissue lyser (Qiagen, Part.No. 20.747.0001) two-times for 2.5 minutes at 30 Hz. Afterwards, 200 µl chloroform were added per sample. Sample tubes were closed and vigorously shaken by hand for 15 sec, incubated for 2-3 min at room temperature, and then centrifuged at 12000 g for 15 min at 4°C. The aqueous phase of each sample was transferred to a new tube, and 500 μl of isopropyl alcohol (isopropanol / 2-propanol) was added. Tubes were gently shaken by turning the tubes upside-down 4-6 times. Afterwards, they were incubated for 10 min at room temperature. Samples were then centrifuged at 12000 g for 10 min at 4°C, and the supernatant was afterwards discarded while RNA formed gel-like pellets within the tubes which were retained. At least 1 ml ethanol (75%, in PCR-grade water) was added to each tube. Samples were then gently mixed and afterwards centrifuged at 7500 g for 5 min at 4°C.

For RNA purification, the RNeasy Mini kit (Qiagen 74104 or 74106) and the RNAase free DNAase kit (Qiagen 792454) were used. Samples were taken out of the -80°C freezer and spun down shortly. The supernatants were discarded and the RNA pellets briefly air-dried for 5-10 min. To dissolve the RNA pellets, two-times 150 µl RNase-free water was added and pipetted up and down a few times. Samples were then incubated two-times for 10 min at 55°C, being flicked in between. Afterwards 600 µl RLT-buffer with 1% β-mercapto ethanol was added to the samples, and the remainder of the pellets was dissolved by pipetting the buffer solution up and down a few times. After spinning down the samples shortly, 600 µl of 70% ethanol was added. Samples were shaken afterwards, and the solutions were transferred to the membrane of a RNeasy spin column, placed within a 2 ml collection tube. After centrifuging (15 min, 8000 g, room temperature), the flow through was discarded. To wash the membranes, 350 µl RW1 buffer was added to the RNeasy spin columns, which were afterwards centrifuged (15 min, 8000 x g, room temperature). Then, 80 µl DNase I incubation mix were added to the membranes, which were thereafter incubated for 15 min at room temperature and afterwards washed again with 350 µl RW1 buffer. For a second washing step, two times 500 µl RPE buffer were added to the columns, and centrifuged at 8000 g for 15 and 2 minutes, respectively. A full-speed centrifugation for 1 min, using new collection tubes, removed remaining buffer. RNA was then placed on the membranes. For the final elution step, two times 50 µl RNA storage solution (Ambion) were added, samples centrifuged for 1 minute at 8000 g, and stored at -80°C for later analysis.
