# Supplementary material for: Effects of adult temperature on gene expression in a butterfly: identifying pathways associated with thermal acclimation
Source: BMC Evol Biol. 2019 Jan 23;19:32. doi: 10.1186/s12862-019-1362-y (PMC6345059; doi:10.1186/s12862-019-1362-y)
Supplement: Supplementary file 6 — Table S2. The table shows the distribution of protein coverage for the Trinity and SOAP assemblies of the Bicyclus anynana transcriptome. Trinity produced a better assembly such that only Trinity was used in further analyses, whereas the SOAP assembly was discarded. (DOCX 13 kb) [file 12862_2019_1362_MOESM6_ESM.docx]

**Additional file6: Table S2**

**Assembly comparison**

Protein coverage distributions for the Trinity and SOAP assemblies of the *Bicyclus anynana* transcriptome, after having run a blastx search against the *Drosophila melanogaster* proteome. % coverage: Percentage of protein coverage given as ranges. Frequency: Number of proteins that aligned within a specific coverage range. For example, in the SOAP assembly 1018 proteins aligned with a coverage between 90 and 100%. Cumulative: Cumulative number of proteins that aligned up to a specified coverage level. For example, in the SOAP assembly 1574 proteins aligned with a coverage of at least 80%.

| **% Coverage** | **SOAP** | | **Trinity** | |  |
| --- | --- | --- | --- | --- | --- |
|  | Frequency | Cumulative | Frequency | Cumulative | |
| 90-100 | 1018 | 1018 | 2670 | 2670 | |
| 80-90 | 556 | 1574 | 1024 | 3694 | |
| 70-80 | 548 | 2122 | 774 | 4468 | |
| 60-70 | 584 | 2706 | 712 | 5180 | |
| 50-60 | 679 | 3385 | 678 | 5858 | |
| 40-50 | 893 | 4278 | 679 | 6537 | |
| 30-40 | 990 | 5268 | 696 | 7233 | |
| 20-30 | 1046 | 6314 | 771 | 8004 | |
| 10-20 | 1004 | 7318 | 761 | 8765 | |
| 0-10 | 0 | 7318 | 0 | 8765 | |
