# Supplementary material for: Effects of adult temperature on gene expression in a butterfly: identifying pathways associated with thermal acclimation
Source: BMC Evol Biol. 2019 Jan 23;19:32. doi: 10.1186/s12862-019-1362-y (PMC6345059; doi:10.1186/s12862-019-1362-y)
Supplement: Supplementary file 7 — Here a justification is given for analysing males and females in a joint analysis. (DOCX 14 kb) [file 12862_2019_1362_MOESM7_ESM.docx]

**Additional file 7**

**Alternative differential expression analysis separated by sex**

A joint analysis of all samples in one model has the potential weakness that an assumption that edgeR makes may be violated. It assumes a negative binomial distribution with one dispersion parameter (that determines the variance given the mean) for each transcript (‘tag’) and does not account for the case that different dispersion parameters may be appropriate for subsets of the samples (specifically females *versus* males). We therefore also performed the analyses for 36 males and 39 females separately. For both subsets we used a GLM of the form

$$\ln\mu_{g,j}= \beta_{1}+ \beta_{2}t_{j}+ \beta_{3}f_{j}+\beta_{4}f_{j}t_{j}$$

and tested against the null hypothesis that there is no effect of temperature ($\beta_{2}=\beta_{4}=0)$. In males and females, 752 and 2227 transcripts were significant, respectively. 200 transcripts were in the intersecting set, i.e. significantly differently expressed with regard to temperature in both males and females. Notably, the set of significantly differentially expressed transcripts in females is much larger than in males. This could be a result of the fact that in females the absolute read counts per transcript are substantially larger (on average 426.5 in females *versus* 345.1 in males). In addition to the somewhat larger sample size of females, this could explain a higher statistical power for female samples. Because a larger set of *significantly* differentially expressed transcripts does not imply that also a larger set of transcripts *is* differentially expressed, this comparison is inconclusive. Moreover, the estimates of the dispersion parameter are very similar in males and females (1.101 versus 1.103). We therefore chose to analyze all samples together and to benefit from the increase in power that is to be expected from this larger, pooled sample of individuals.
