# Supplementary material for: Effects of adult temperature on gene expression in a butterfly: identifying pathways associated with thermal acclimation
Source: BMC Evol Biol. 2019 Jan 23;19:32. doi: 10.1186/s12862-019-1362-y (PMC6345059; doi:10.1186/s12862-019-1362-y)
Supplement: Supplementary file 9 — This file gives an overview of the results based on gene ontology terms. The assembled transcript clusters were annotated against both, cluster of orthologous groups/eukaryotic orthologous groups (COG/KOG) as well as gene ontology terms. As both annotations gave very similar results, we only present the results based on the COG/KOG system in the main text. (DOCX 461 kb) [file 12862_2019_1362_MOESM9_ESM.docx]

**Additional file 9**

**Gene expression patterns based on Gene Ontology terms**

UniProt was used to assign assembled transcript clusters to gene ontology (GO) terms (<http://geneontology.org/>). The GO classification system distinguishes between three classifications into biological processes, molecular functions, and cellular components. A Venn diagram based on GO annotations showed that the vast majority of expression changes was induced by the factor sex (Fig. 1). Out of the 6497 significantly differentially expressed transcript clusters (p < 0.05), 1178 could be assigned to gene ontology terms. Annotation rates were 18.0% for the factor sex, 22.0% for temperature, and 34.5% for feeding regime. For temperature, we could retrieve 78 biological processes and 123 molecular functions, for sex 341 biological processes and 390 molecular functions, and for feeding 7 biological process and 11 molecular functions.

Out of the 271 annotated transcripts differentially expressed between the temperatures, 191 were down-regulated and 80 were up-regulated at the higher temperature. According to the 83 (out of the above 191) downregulated transcripts related to known biological processes, the most prominent downregulated processes included carbohydrate metabolic processes (5.6%), chitin metabolic processes (5.6%), protein folding (5.6%), and translation (4.6%; Fig. 2a). Regarding molecular functions (n = 120 transcripts with known functions), those most strongly down-regulated at the higher temperature were ATP binding (12.0%), DNA binding (4.7%), chitin binding, metal ion binding, ribosomal and threonine-type endopeptidase activity (all 3.1%; Fig. 2e). Genes strongly down-regulated at the higher temperature were a DNA cross-link repair and two cuticular proteins (Fig. 3a). Biological processes upregulated at the higher temperature included chitin metabolic processes (33.3%), lipid metabolic processes, and ribosome biogenesis (both 6.1%; Fig. 2b). Up-regulated molecular functions included chitin binding (13.0%), calcium-release channel activity (5.9%), ATP binding (4.7%), and serine-type endopeptidase activity (4.7%; Fig. 2f). The genes most strongly up-regulated at the higher temperature were V-type proton ATPase subunit C, a probable methylmalonate-semialdehyde dehydrogenase, and furin-like convetase (Fig. 3a).

Out of the 2149 annotated transcripts that were differentially expressed among both sexes, 1260 were down- and 889 up-regulated in females relative to males. Out of the above, in total 363 transcripts were related to known biological processes. Biological processes down-regulated in females were frequently related to chitin metabolic processes (12.6%), metabolic processes (5.4%), and carbohydrate metabolic processes (4.6%; Fig. 2c). Molecular functions were known for 1370 out of the 2149 annotated transcripts. The molecular functions being most strongly down-regulated in females were chitin binding (6.6%), ATP binding (5.1%), metal ion binding (3.3%), and oxidoreductase activity (2.9%; Fig. 2g). The most strongly down-regulated genes in females were immulectin and enolase (Fig. 3b). Biological processes being up-regulated in females relative to males included regulation of transcription (4.7%), transcription (4.5%), chitin metabolic processes, and DNA replication (both 2.6%; Fig. 2d). Molecular functions that were up-regulated in females included ATP binding (11.6%), DNA binding (5.8%), and zinc ion binding (4.7%; Fig. 2h). The most strongly up-regulated proteins in females were a hox cluster protein and a seminal fluid protein (Fig. 3b).

Only ten transcripts were differentially expressed between feeding treatments, eight of which were down-regulated and two were up-regulated under control conditions. Only three of the down-regulated transcripts could be assigned to a biological process, viz. regulation of transcription, protein glycosylation, and protein folding. For five transcripts a molecular function was found, viz. pyruvate kinase activity, DNA binding, ATP binding, glycotransferase activity, and unfolded protein binding. For both transcripts being upregulated under control conditions, we found according biological processes, namely regulation of translation and nucleoside metabolic processes. The respective molecular functions were RNA and magnesium ion binding.


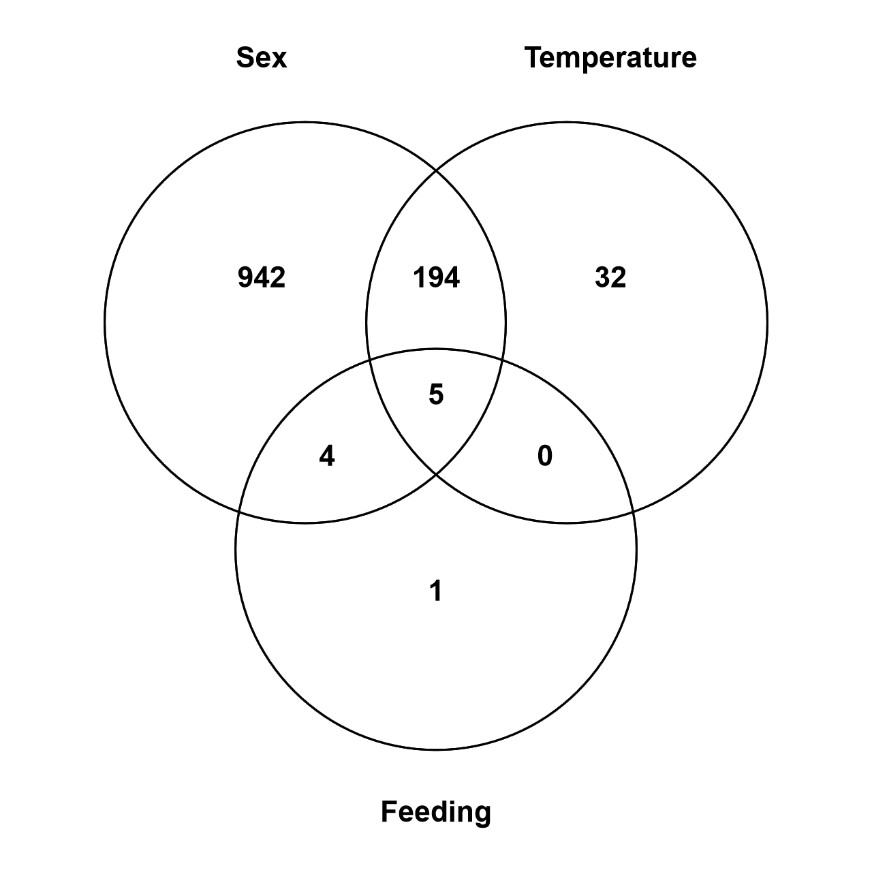


**Fig. 1:** Venn diagram depicting the patterns of private and shared genes among the factors sex, temperature, and feeding regime for 1178 differentially expressed transcript clusters based on Gene Ontology (GO) terms.

| 1: Carbohydrate metabolic process |
| --- |
| 2: Chitin metabolic process |
| 3: Protein folding |
| 4: Translation |
| 5: Glycolytic process |
| 6: ATP hydrolysis coupled proton transport |
| 7: DNA recombination |
| 8: DNA repair |
| 9: DNA replication |
| 10: Response to stress |
| 11: Others |


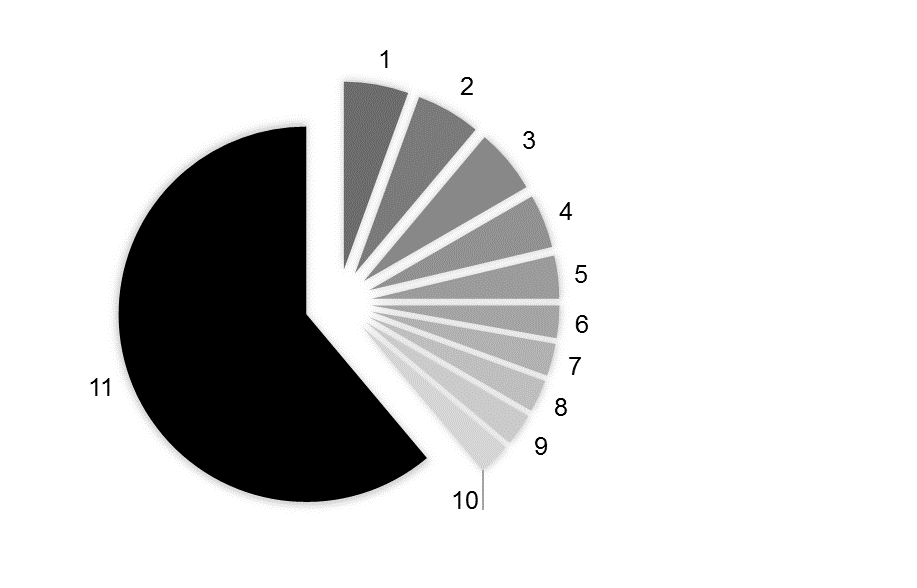


Fig. 2a

| 1: Chitin metabolic process |
| --- |
| 2: Lipid metabolic process |
| 3: Ribosome biogenesis |
| 4: ATP hydrolysis coupled proton transport |
| 5: Feeding behavior |
| 6: Formation of translation preinitiation complex |
| 7: Lipid transport |
| 8: Metabolic process |
| 9: Neuropeptide signaling pathway |
| 10: Others |


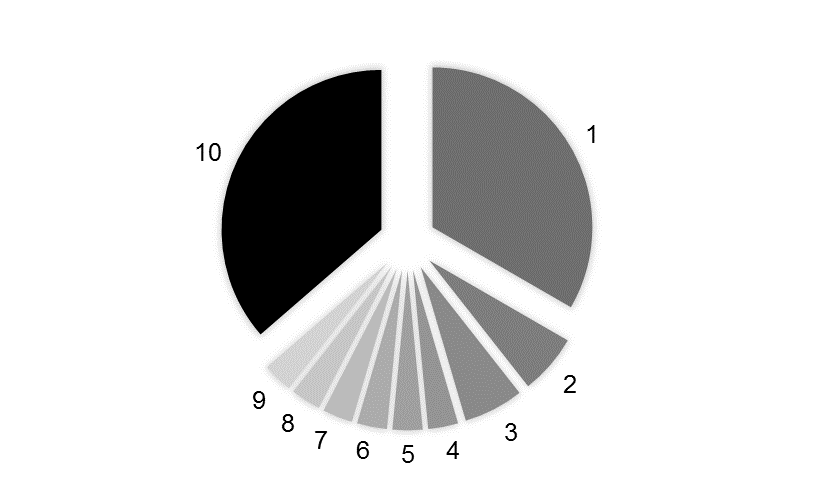


Fig. 2b

| 1: Chitin metabolic process |
| --- |
| 2: Metabolic process |
| 3: Carbohydrate metabolic process |
| 4: Wnt signaling pathway |
| 5: Translation |
| 6: Lipid metabolic process |
| 7: Microtubule-based process |
| 8: Transmembrane transport |
| 9: ATP hydrolysis coupled proton transport |
| 10: Glycolytic process |
| 11: Fructose 6-phosphate metabolic process |
| 12: Regulation of transcription, DNA-templated |
| 13: Transcription, DNA-templated |
| 14: Allantoin catabolic process |
| 15: Angiotensin maturation |
| 16: Carboxylic acid metabolic process |
| 17: Fatty acid beta-oxidation |
| 18: Immune response |
| 19: Tricarboxylic acid cycle |
| 20: ATP synthesis coupled proton transport |
| 21: Neuropeptide signaling pathway |
| 22:Oxidation-reduction process |
| 23:Others |


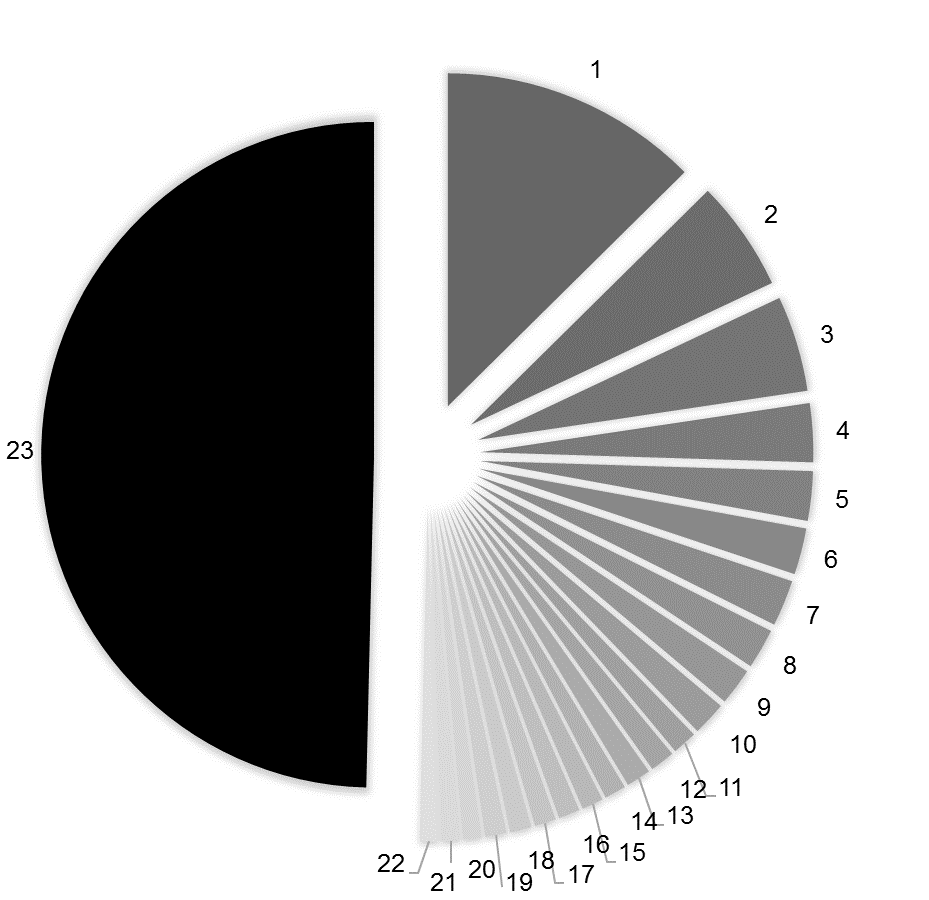


Fig. 2c

| 1: Regulation of transcription, DNA-templated |
| --- |
| 2: Transcription, DNA-templated |
| 3: Chitin metabolic process |
| 4: DNA replication |
| 5: DNA repair |
| 6: Protein folding |
| 7: Translation |
| 8: Multicellular organism development |
| 9: DNA recombination |
| 10: mRNA processing |
| 11: Protein transport |
| 12: RNA processing |
| 13: Small GTPase mediated signal transduction |
| 14: Cell division |
| 15: Intracellular protein transport ] |
| 16: Microtubule-based process |
| 17: tRNA processing |
| 18: Ubiquitin-dependent protein catabolic process |
| 19: Others |


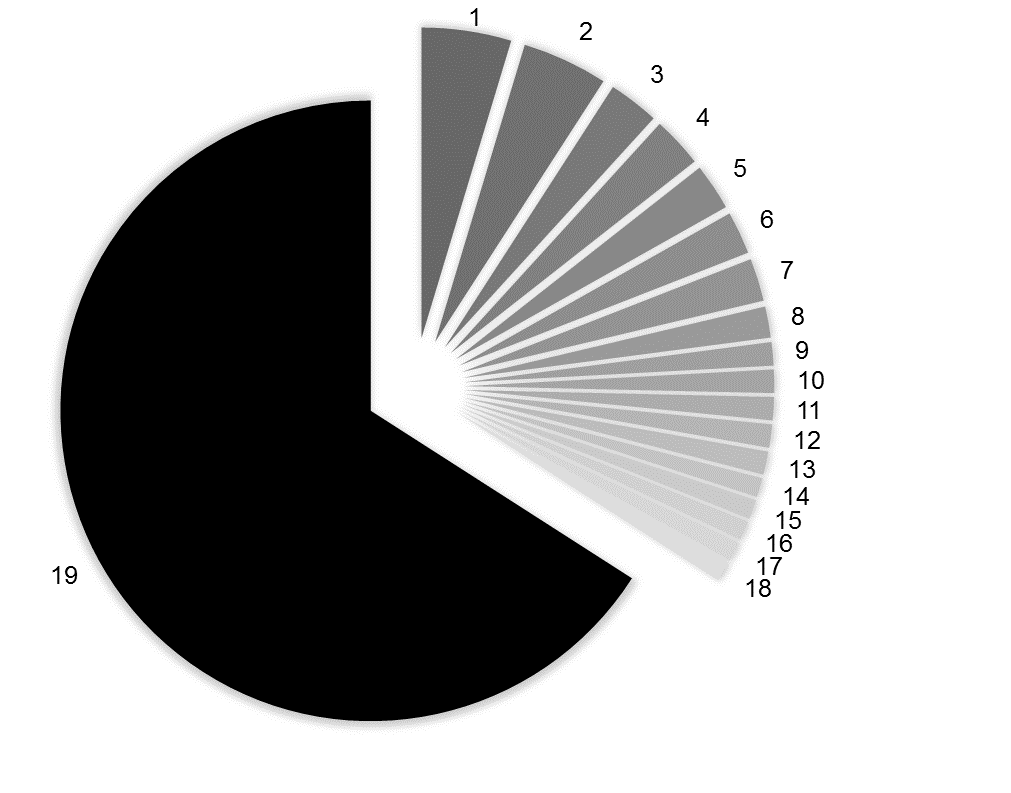


Fig. 2d

| 1: ATP binding |
| --- |
| 2: DNA binding |
| 3: Chitin binding |
| 4: Metal ion binding |
| 5: structural constituent of ribosome |
| 6: Threonine-type endopeptidase activity |
| 7: Motor activity |
| 8: RNA binding |
| 9: Unfolded protein binding |
| 10: GTP binding |
| 11: Actin filament binding |
| 12: Catalytic activity |
| 13: GTPase activity |
| 14: Hydrogen ion transmembrane transporter activity |
| 15: Nucleic acid binding |
| 16: Oxidoreductase activity |
| 17: Serine-type endopeptidase activity |
| 18: Zinc ion binding |
| 19: Others |

Fig. 2e

| 1: Chitin binding |
| --- |
| 2: Ryanodine-sensitive calcium-release channel activity |
| 3: ATP binding |
| 4: Serine-type endopeptidase activity |
| 5: Heme binding |
| 6: Adrenergic receptor activity |
| 7: Calcium ion binding |
| 8: DNA binding |
| 9: Metal ion binding |
| 10: Metal ion binding |
| 11: Zinc ion binding |
| 12: Oxidoreductase activity |
| 13: Others |


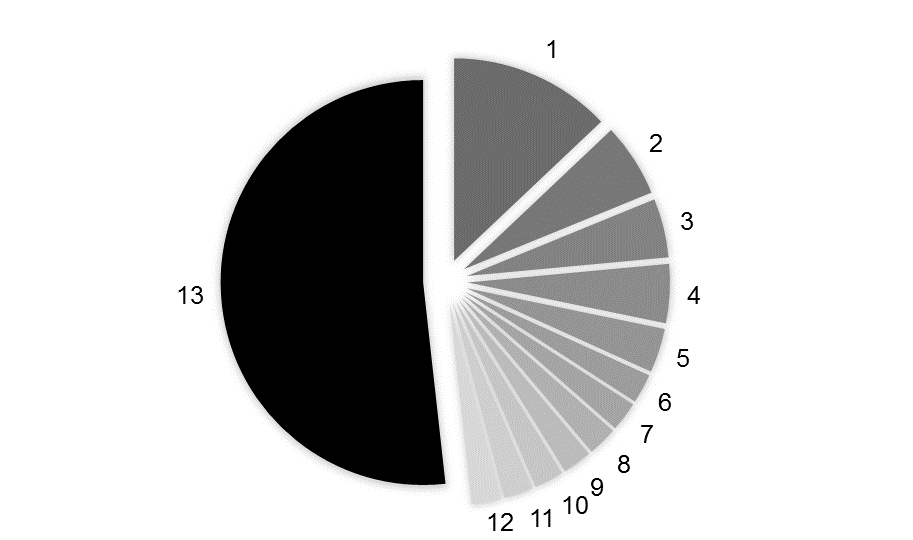


Fig. 2f

| 1: Chitin binding |
| --- |
| 2: ATP binding |
| 3: Metal ion binding |
| 4: Oxidoreductase activity |
| 5: Serine-type endo-peptidase activity |
| 6: Zinc ion binding |
| 7: Calcium ion binding |
| 8: Heme binding |
| 9: Hydrolase activity |
| 10: Motor activity |
| 11: Carbohydrate binding |
| 12: Iron ion binding |
| 13: Glucuronosyl-transferase activity |
| 14: Lipid binding |
| 15: DNA binding transcription factor activity |
| 16: Actin filament binding |
| 17: GTP binding |
| 18: Lipid transporter activity |
| 19: Oxidoreductase activity |
| 20: Others |


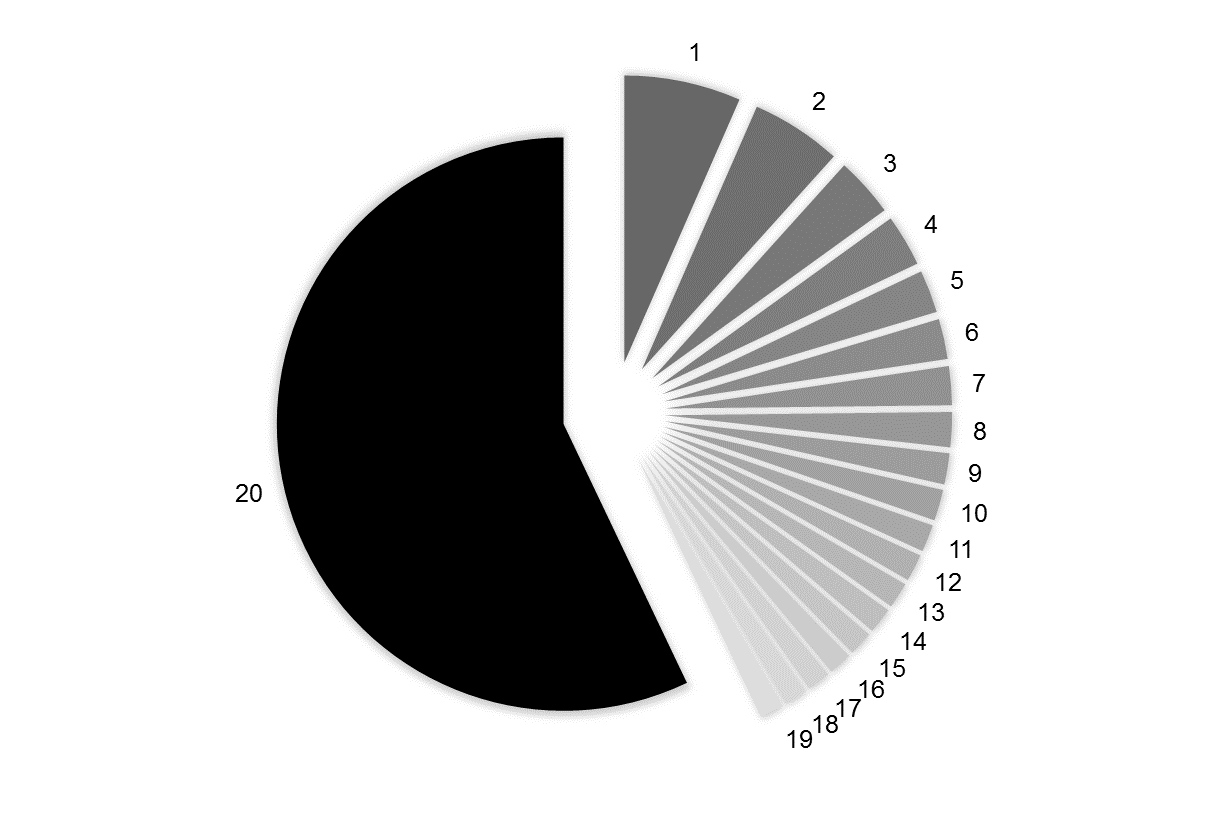


Fig. 2g

| 1: ATP binding |
| --- |
| 2: DNA binding |
| 3: Zinc ion binding |
| 4: RNA binding |
| 5: Nucleic acid binding |
| 6: GTP binding |
| 7: Metal ion binding |
| 8: GTPase activity |
| 9: Protein serine/threonine kinase activity |
| 10: Sequence-specific DNA binding |
| 11: Chitin binding |
| 12: Helicase activity |
| 13: Protein kinase activity |
| 14: Calcium ion binding |
| 15: Structural constituent of ribosome |
| 16: Unfolded protein binding |
| 17: Methyltransferase activity |
| 18: DNA binding transcription factor activity |
| 19: Heme binding |
| 20: Peptidase activity |
| 21: Translation initiation factor activity |
| 22: Others |


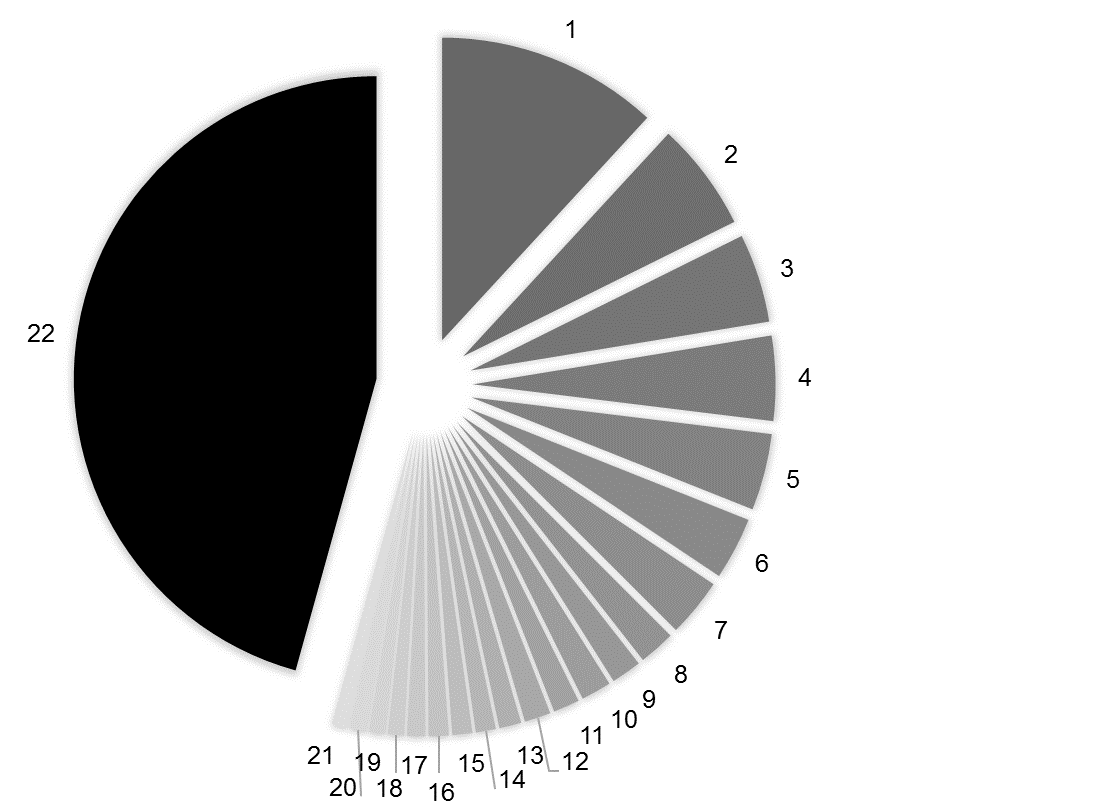


Fig. 2h

**Fig. 2:** Functional annotation of transcripts to biological processes being (a) down- or (b) up-regulated at the higher temperature, and (c) down- or (d) up-regulated in females relative to males, and functional annotation of transcripts to molecular functions being (e) down- or (f) up-regulated at the higher temperature, and (g) down- or (h) up-regulated in females relative to males.


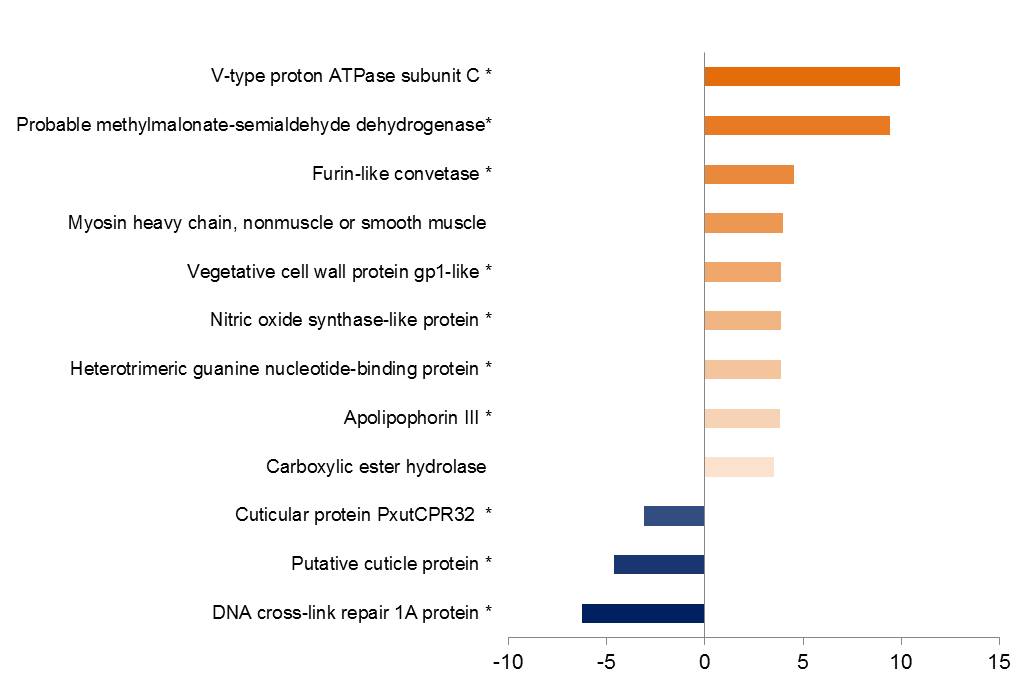


Fig. 3a


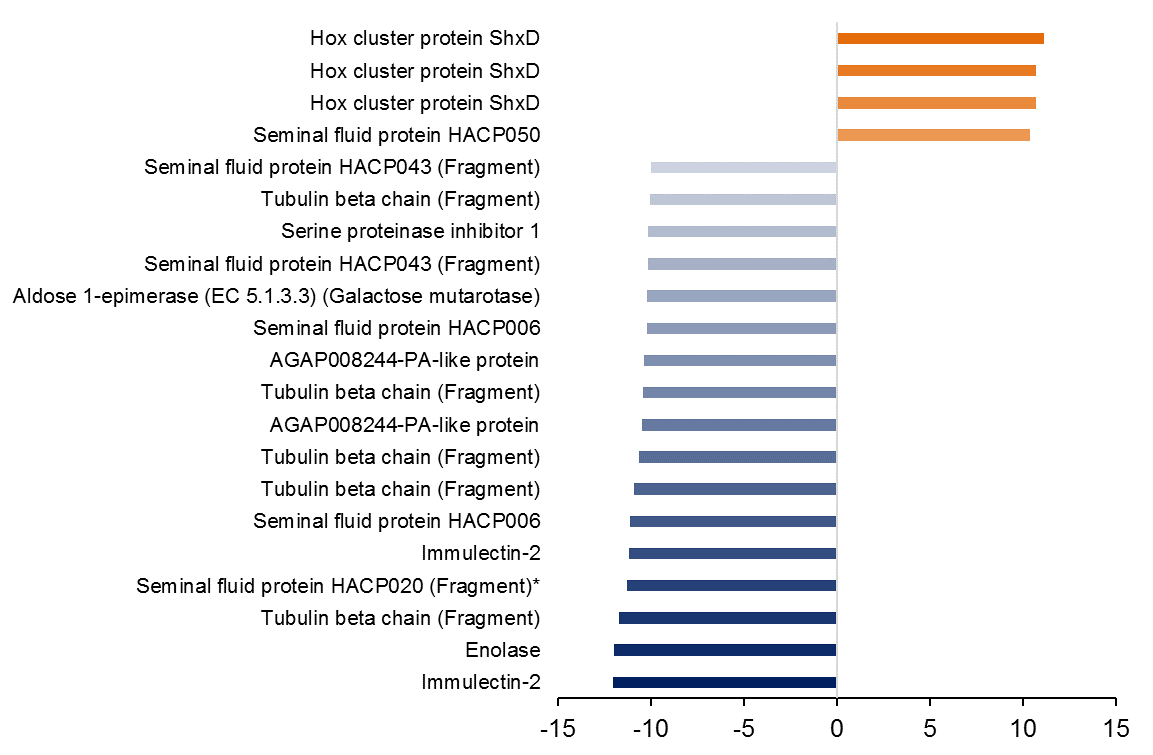


Fig. 3b

**Fig. 3:** Overview of genes in which expression was most strongly affected by the factors (a) temperature and (b) sex. In (a) genes with logFC values below -3 or above +3 and in (b) below -10 or above +10 are shown. Blue: down-regulation at higher temperature and in females; red: up-regulation at higher temperature and in females. The darker the colour the stronger the up- or down-regulation. Genes marked by * are private genes.
